# Item Response Theory Modeling of the International Prostate Symptom Score in Patients with Lower Urinary Tract Symptoms Associated with Benign Prostatic Hyperplasia

**DOI:** 10.1208/s12248-020-00500-w

**Published:** 2020-08-27

**Authors:** Yassine Kamal Lyauk, Daniël M. Jonker, Trine Meldgaard Lund, Andrew C. Hooker, Mats O. Karlsson

**Affiliations:** 1 Translational Medicine, Ferring Pharmaceuticals A/S, Kay Fiskers Plads 11, 2300 Copenhagen, Denmark; 2 Department of Drug Design and Pharmacology, University of Copenhagen, Copenhagen, Denmark; 3 Department of Pharmaceutical Biosciences, Uppsala University, Uppsala, Sweden

**Keywords:** item response theory, BPH, LUTS, International Prostate Symptom Score, pharmacometrics

## Abstract

Item response theory (IRT) was used to characterize the time course of lower urinary tract symptoms due to benign prostatic hyperplasia (BPH-LUTS) measured by item-level International Prostate Symptom Scores (IPSS). The Fisher information content of IPSS items was determined and the power to detect a drug effect using the IRT approach was examined. Data from 403 patients with moderate-to-severe BPH-LUTS in a placebo-controlled phase II trial studying the effect of degarelix over 6 months were used for modeling. Three pharmacometric models were developed: a model for total IPSS, a unidimensional IRT model, and a bidimensional IRT model, the latter separating voiding and storage items. The population-level time course of BPH-LUTS in all models was described by initial improvement followed by worsening. In the unidimensional IRT model, the combined information content of IPSS voiding items represented 72% of the total information content, indicating that the voiding subscore may be more sensitive to changes in BPH-LUTS compared with the storage subscore. The pharmacometric models showed considerably higher power to detect a drug effect compared with a cross-sectional and while-on-treatment analysis of covariance, respectively. Compared with the sample size required to detect a drug effect at 80% power with the total IPSS model, a reduction of 5.9% and 11.7% was obtained with the unidimensional and bidimensional IPSS IRT model, respectively. Pharmacometric IRT analysis of the IPSS within BPH-LUTS may increase the precision and efficiency of treatment effect assessment, albeit to a more limited extent compared with applications in other therapeutic areas.

**Electronic supplementary material:**

The online version of this article (10.1208/s12248-020-00500-w) contains supplementary material, which is available to authorized users.

## INTRODUCTION

Benign prostate hyperplasia (BPH) is a common condition in the aging male and is estimated to affect 50% of males by age 60 years and 90% by age 85 years (1,2). The clinical manifestations of BPH are known as lower urinary tract symptoms (LUTS) and are characterized by an increased: sensation of incomplete emptying of the bladder following urination, urination frequency, urination intermittency, urgency to urinate, weakness of the urinary stream, straining to start urination, and nocturia. LUTS are associated with adverse health effects such as significantly diminished quality of life and depression, as well as impairment in activities of daily living (3–5). In approximately 10% of patients, the condition may lead to severe complications such as acute urinary retention, urosepsis, and kidney failure (2,6). The severity of BPH-LUTS is commonly measured by the International Prostate Symptom Score (IPSS) (also known as the American Urological Association score) (7), which consists of seven questions describing the severity of each of the clinical manifestations of LUTS. The IPSS questionnaire is considered the gold standard measure for assessing BPH-LUTS, and its use is widespread in the clinic, as a primary or secondary endpoint in clinical trials, and in urology research (8).

Pairwise cross-sectional testing based on the summary score mean change from baseline is the traditional pre-specified analysis for clinical trials using scale measures as the primary efficacy endpoint. However, analysis of clinical trial data through longitudinal pharmacometric modeling has been shown to increase the power to detect a drug effect compared with pairwise testing (9–11). Furthermore, an extension of longitudinal pharmacometric modeling specific to multiple-item questionnaire data (9), which utilizes concepts derived from item response theory (IRT), has identified the potential for increased assessment precision in several therapeutic areas (namely, Alzheimer’s disease, Parkinson’s disease, multiple sclerosis, and depression) (9,12–14). Moreover, the methodology has shown an increase in the power to detect a drug effect compared with longitudinal pharmacometric analysis of summary score data (9,15). Briefly, IRT quantifies the relationship between an individual’s intrinsic trait (e.g., disability) and the probability of answering a questionnaire (e.g., IPSS) in a particular way (16,17). By preserving the information contained within responses to individual items, it is possible to estimate an individual’s latent disability, how well items discriminate between individuals with differing estimates of latent disability, and the location of item responses along the disability scale.

The GnRH receptor antagonist, degarelix, approved for the treatment of advanced prostate cancer (Firmagon®), was investigated as an alternative medical approach for the treatment of moderate-to-severe BPH-LUTS in patients without prostate cancer. Due to its depot formation upon administration, functioning as a slow-release formulation, treatment with degarelix was envisioned to achieve greater compliance and effectiveness compared with currently approved treatments requiring daily administration. The degarelix doses tested within BPH-LUTS were substantially lower than the approved doses used for treating prostate cancer (a loading dose of 240 mg followed by maintenance doses of 80 mg) to avoid eliciting prolonged testosterone suppression in patients.

To date, only one publication describes longitudinal model–based analysis of the total IPSS (18) and, moreover, longitudinal pharmacometric IRT modeling has not been applied to the analysis of the IPSS within BPH-LUTS. Using data from 403 patients in a phase II trial investigating the treatment of moderate-to-severe BPH-LUTS with degarelix over 6 months, we set out to (i) characterize the internal characteristics of the IPSS through IRT analysis of the item-level data, (ii) utilize the obtained IRT information to develop pharmacometric IRT models describing the time course of underlying BPH-LUTS, and (iii) examine the power to detect a drug effect of pharmacometric IRT IPSS modeling compared with cross-sectional testing and longitudinal modeling, respectively, based on total IPSS.

## METHODS

### Data

The IPSS is a seven-item questionnaire, where each item can be scored from 0 to 5, yielding a composite IPSS ranging from zero to 35. Item scores reflect symptom frequency (not at all, less than 1 in five times, less than half the time, about half the time, more than half the time, and almost always) except for the nocturia item, where they correspond to categorized counts (0 to ≥ 5 awakenings).

Ferring Pharmaceuticals’ A/S trial CS36 (NCT00947882) was a phase II, double-blind, parallel-group, dose-finding study evaluating the efficacy and safety of degarelix over 6 months. Following a wash-out period, 403 patients were randomized to a single subcutaneous injection of 10, 20, or 30 mg degarelix 40 mg/mL solution, or placebo and were required to have an IPSS ≥ 13 at screening 2 weeks prior to dosing at the baseline visit. The primary endpoint was the mean change from baseline in IPSS compared with placebo 3 months after dosing. Visits were planned at 2 weeks, and 1, 2, 3, 4, 5, and 6 months after dosing. Rich pharmacokinetic sampling (*n* = 15) was performed in 43 patients while sparse (*n* = 2) pharmacokinetic sampling was performed in 240 patients. An interim trial analysis was planned for 6 months post-dosing in order to stop the trial early if the primary endpoint was not met. Trial CS36 was conducted in accordance with the Declaration of Helsinki and Good Clinical Practice.

### Item Response Theory Modeling

The score for each of the seven IPSS items may range from zero to five. The relationship between disability and the probability (*P*) of a patient answering a score of at least *k* was therefore modeled through a graded response model (19):

$$ P\left({Y}_{ij}\ge k\right)=\frac{e^{a_{j\kern0.5em }\left({\psi}_i-{b}_{j.k}\right)}}{1+{e}^{a_{j\kern0.5em }\left({\psi}_i-{b}_{j.k}\right)}} $$

where *Y*_*ij*_ represents the score of patient *i* on item *j*, *a*_*j*_ the slope/discrimination parameter of item *j*, *ψ*_*i*_ the unobserved disability of patient *i*, and *b*_*j*_ the difficulty parameter of item *j*. Cumulative probabilities for an item with a score of maximum 5 were modeled as follows:

$$ {\displaystyle \begin{array}{c}P\left({Y}_{ij}=0\right)=1-P\left({Y}_{ij}\ge 1\right)\\ {}P\left({Y}_{ij}=k\right)=P\left({Y}_{ij}\ge k\right)-P\left({Y}_{ij}\ge k+1\right)\\ {}P\left({Y}_{ij}=5\right)=P\left({Y}_{ij}\ge 5\right)\end{array}} $$

Item characteristic curves (ICCs) were estimated as fixed effects by treating IPSS measurements from each patient’s study visit as originating from a separate individual (in this work referred to as the *IDVIS* approach). Disability was estimated as a random effect, and its distribution was fixed to a standard normal distribution (mean 0 and variance 1) at baseline. Post-baseline shift parameters were included to allow for a different mean and variance of disability post-baseline (where disability is likely to have changed compared with baseline due to placebo and/or drug effects). A similar ICC estimation approach has been reported previously in the literature (13,14,20,21).

Factor analysis (FA) is an established statistical method (22) for assessing item patterns and informing the item structure of IRT models (23). The procedure is aimed at explaining the interrelationship between many observed variables by way of few latent variables and is based on analysis of the between-item correlation matrix. It may be used to identify the number of questionnaire domains and identify which items correspond to each of these (exploratory FA) or to investigate the item patterns with a pre-specified number of factors (confirmatory FA). Lastly, it may also inform whether the assumption of only one general dimension for all items is supported (24). In the current work, a unidimensional IRT model was first fit to the CS36 data, and the adequacy of the unidimensionality assumption was assessed based on the item factor loadings. The latter indicate an item’s correlation with the factor, where higher absolute values suggest closer association. Following development of the unidimensional IRT model, confirmatory FA with two dimensions (a minimum of three items per dimension is needed to preserve model identification) and varimax orthogonal rotation (25) was used to inform the item structure of a bidimensional IRT model. In the developed IRT ICC models, residual correlation between items was also assessed and was calculated as follows:

$$ {\displaystyle \begin{array}{c}{\mathrm{RES}}_{ij}={\mathrm{DV}}_{ij}-{E}_{ij}\\ {}{E}_{ij}=P(1)\ast 1+P(2)\ast 2+P(3)\ast 3+P(4)\ast 4+P(5)\ast 5\end{array}} $$

with DV_ij_ being the observed score from the *i*th individual for the *j*th IPSS item and *E*_ij_ being the corresponding weighted prediction based on the IRT-derived ICCs and individual disability estimates.

#### Pharmacometric Implementation of Item Response Theory

Following the IRT ICC estimation step, the resulting knowledge was incorporated into a pharmacometric framework. First, the original individual assignment was reconciled with the data (i.e., longitudinal observations were restored for each patient), and IRT-derived latent disability estimates were modeled longitudinally as the dependent variable. Uncertainty in the Empirical Bayes Estimates (EBEs) of latent disability was taken into account through an additional additive residual error model term, similar to the IPPSE (individual PK parameters with standard errors) approach in sequential PK/PD modeling (26) (we here name it the PSI-IPPSE approach). Schindler *et al.* previously proposed a similar approach (20) but without standard errors. Secondly and lastly, the IRT ICC estimation model and the final longitudinal latent disability model from the PSI-IPPSE step were combined into a single model to allow translation of latent disability to observed IPSS at the item and summary level, respectively. In the latter model, the impact of re-estimating only the longitudinal parameters, as well as the simultaneous estimation of ICCs and longitudinal parameters, was examined.

#### Calculation of Fisher Information Content

To investigate which IPSS items carry the most information (i.e., the signal-to-noise ratio in determining patients’ latent disability) and where on the disability scale they are most informative, the Fisher information content of each IPSS item was calculated as the negative expectation of the second derivative of the log-likelihood using the unidimensional IRT ICC estimation model. The information functions were visualized to illustrate the sensitivity of each IPSS item over the full disability range. Individual items were ranked according to the amount of information they contained relative to the total information based on each item’s calculated area under the curve within this study’s estimated disability range. Information content assessment was performed in the context of unidimensional IRT modeling. This allows for an overall perspective across all IPSS items while in the multidimensional IRT framework, it is only feasible within each separate dimension.

### Structural Longitudinal Modeling

For underlying disability in the context of IRT as well as observed total IPSS, a similar approach to longitudinal model development was undertaken. First, data from patients randomized to the placebo group were modeled. Here, different structural models were tested to best describe the time course of the placebo effect, such as linear, bi-linear, power, exponential, Weibull, Gompertz, and inverse Bateman models. The addition of a linear drift parameter (27) to describe worsening or continued improvement was tested for all abovementioned models. Subsequently, data from patients assigned to degarelix treatment were added to the data set to describe the drug effect. In this step, we investigated models describing degarelix treatment effects as present or absent, independent of the administered dose, as well as dose-response models (linear and Emax). An offset treatment effect, as well as onset treatment effects to describe time delays in reaching the full response (linear, exponential, slope-intercept models), was investigated. Normally and log-normally distributed between-subject variability was investigated for all parameters. For the total IPSS model, additive, proportional, and combined error models were investigated to describe residual variability.

### Covariate Analysis

Investigated baseline covariates consisted of demographics (age, weight, and body mass index), physiological disease-specific measures (total prostate volume, serum testosterone, prostate-specific antigen, average flow rate, flow time including time to maximum flow, maximum urine flow, post-void residual volume, voiding time, and voiding volume), validated disease-specific patient-reported outcome (quality of life (QoL) score, BPH Impact Index (BII) score), and study site region (North America or Europe). Baseline IPSS was tested as a covariate on the drug effect parameter during longitudinal IPSS modeling. Lastly, individual degarelix area under the curve (AUC_0-∞_) estimates derived from application of a previously developed population pharmacokinetic model (28) to the CS36 trial pharmacokinetic data were investigated as a predictor of treatment effect variability, both as a continuous value and binned by quartile.

Covariate analysis was performed by way of a stepwise search at a significance level of 0.01 in the forward inclusion step and 0.001 in the backward elimination step. Linear relationships were investigated for covariates. A multiplicative covariate model (Eq. 1) was used to test continuous covariates on parameters except in the case of parameters liable to assume a typical value (*θ*) of zero (e.g., baseline disability in longitudinal IRT modeling), where an additive covariate model was used (Eq. 2)

1 $$ \mathrm{Parameter}={\theta}_{\mathrm{Parameter}}\ast \left(1+{\theta}_{\mathrm{Covariate}}\ \left(\mathrm{Covariate}-{\mathrm{Covariate}}_{\mathrm{median}}\right)\right)\kern3.75em $$

2 $$ \mathrm{Parameter}={\theta}_{\mathrm{Parameter}}+{\theta}_{\mathrm{Covariate}\kern0.5em }\left(\mathrm{Covariate}-{\mathrm{Covariate}}_{\mathrm{median}}\right) $$

### Model Evaluation and Diagnostics

Non-covariate–related model selection was based on several criteria: for hierarchal models, the difference in objective function value (OFV) corresponding to a significance level of 0.05 was considered statistically significant assuming a *χ*^2^ distribution while for non-nested models, the difference in Akaike information criterion (AIC) was used. Moreover, model stability based on the convergence of minimization and covariance steps, parameter precision assessed through NONMEM’s relative standard error estimate, and graphical diagnostics were also considered during model selection.

Visual predictive checks (VPCs) of the longitudinal IPSS, as well as the change in IPSS from baseline stratified by treatment arm using 200 samples, were used to assess the adequacy of the model characterization of the observed IPSS data.

In the IRT analyses, the goodness of fit of ICCs was assessed using a novel sampling-based cross-validated generalized additive model (GAM) cubic spline smooth, which builds upon the commonly used GAM smooth diagnostic (21). As for all pharmacometric model diagnostics, EBE-based visual representations may be misleading due to *η*-shrinkage (29). In this particular diagnostic, EBE-shrinkage can cause an adequate model to appear inadequate, in particular at extreme disability values. In order to counteract the potential effects of *η*-shrinkage of disability EBEs on the GAM smooth diagnostic, an approach was developed utilizing random sampling from the individual posterior *η* distributions from the final ICC estimation model uncertainty estimate of EBEs (Fisher information assessed variance or conditional variance). Two hundred *η* samples were drawn randomly, assuming normal distributions with mean individual posterior *η* estimate and variance individual *η* Fisher information assessed variance. Disability estimates were subsequently calculated for each generated *η* while respecting the baseline or post-baseline IDVIS origin of *η*, using the estimated fixed-effects post-baseline shift parameters. Similar to the traditional IRT GAM diagnostic, GAM smooths were applied to the data (one for each unique item–difficulty category combination). To adjust for the difference between the number of sampling-generated and number of actual study–derived disability estimates, the 95% confidence interval of the GAM smooths was adjusted by multiplying the computed standard error with the square root of the number of generated *η* samples. To diagnose the final longitudinal IRT model, VPCs were generated for both item-level IPSS observations and summary IPSS scores using 2000 Monte Carlo simulations.

### Power Calculations

A stochastic simulation and estimation (SSE) procedure with 1000 samples was used to assess the 80% power to detect a drug effect at a 5% level of significance. The model with the lowest AIC among the two developed longitudinal IRT models (unidimensional and bidimensional) was chosen as the simulation model. For simplicity, the Monte Carlo simulations assumed no missing individual IPSS item scores and no drop-out over the 6-month period. Power curves were generated by estimating the power of the models at four different sample sizes, which were informed by an initial exploratory Monte Carlo Mapped Power (MCMP) (30) procedure. In the pharmacometric models, the actual type I error level and corresponding empirically derived ∆OFV was estimated by simulating 1000 trials with no drug effect at each sample size, similar to Wählby *et al.* (31). The power of two different analysis of covariance (ANCOVA) tests was determined using the same simulated data sets on which the power of the pharmacometric models was estimated. Both analyses included treatment as factor and baseline summary IPSS as a covariate. The first ANCOVA used cross-sectional data, regarding only the change from baseline at 3 months post-dose, which was the landmark time point in the CS36 trial. This type of analysis is commonly pre-specified as the main analysis of clinical trials. In the second ANCOVA, the average summary IPSS change from baseline during the entire treatment period was considered the dependent variable, which is known as the “while on treatment” (WOT) strategy/estimand (32). At each sample size, power was determined as the proportion of analyses that identified a statistically significant (*p* < 0.05) treatment effect.

### Software

The Laplacian method in NONMEM version 7.4.3 (33) was used for IRT ICC estimation and final longitudinal IRT modeling, while the first-order conditional estimation with interaction was used for longitudinal IPSS modeling as well as intermediate longitudinal IRT modeling of EBEs of disability. The mIRT R-package (34) version 1.32.0 was used to obtain initial estimates for the ICCs and to perform factor analysis as well as multidimensional IRT model exploration. ICC diagnostics were obtained using R version 4.6.0. Simulation-based model diagnostics for the longitudinal models were obtained using Perl-Speaks-NONMEM (35) (PsN) version 4.9.0.

## RESULTS

**Fig. 1:**
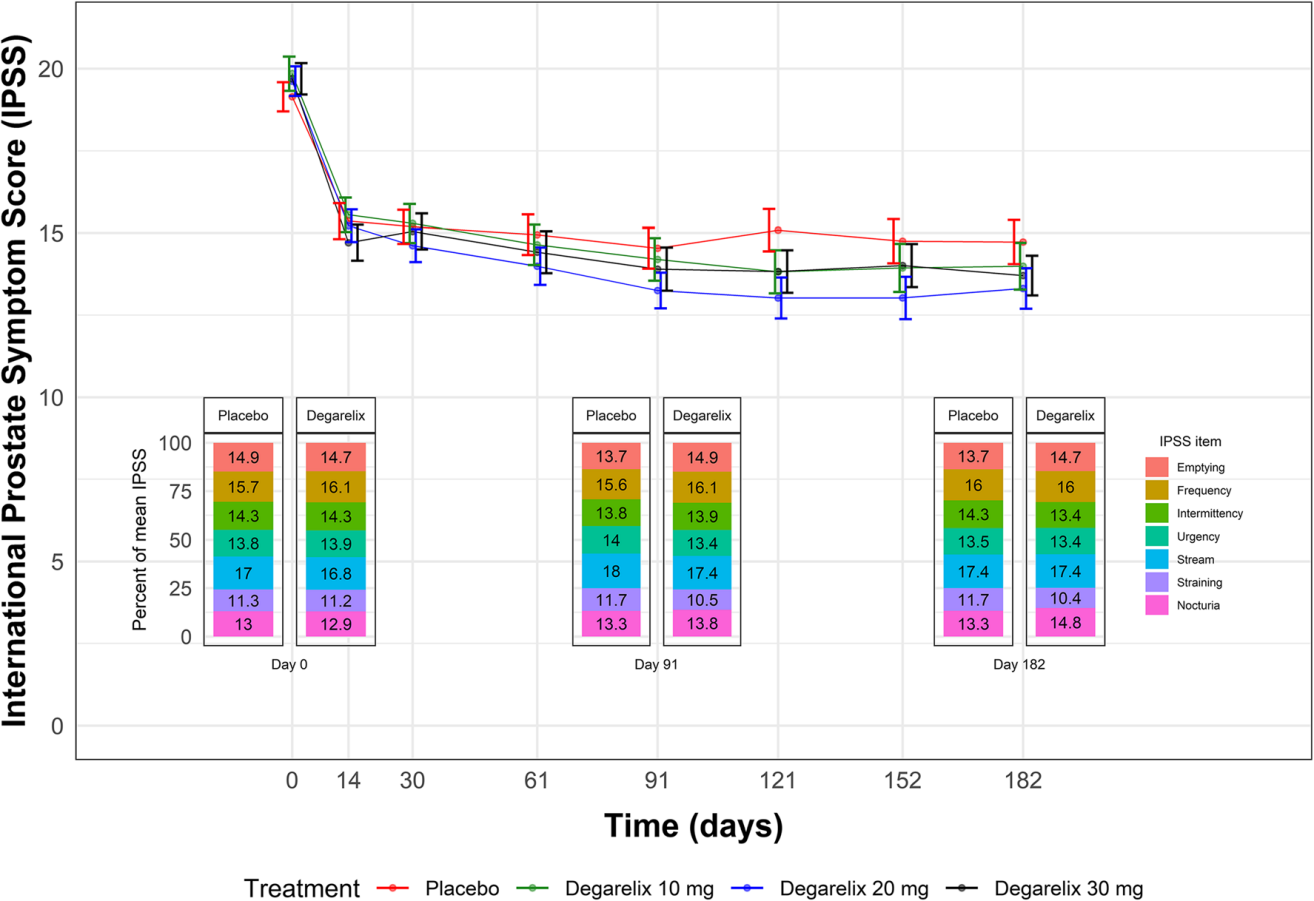
The mean International Prostate Symptom Score (IPSS) in each CS36 trial arm along with the standard error of the mean at each visit. The distribution of item-level IPSS at the baseline visit, landmark time point (3 months post-dose), and end of trial (6 months post-dose) is shown for the placebo arm as well as the pooled degarelix dose arms

Table I shows the subject characteristics at baseline. In total, 3117 summary IPSS and 21,836 item-level IPSS responses from 403 patients were available for analysis. The distribution of responses is shown in Supplemental Fig. S1. Three hundred and sixty-nine of the 403 randomized patients completed the 6-month treatment period. Figure 1 shows the mean summary IPSS time course in each trial arm as well as the distribution of responses for each IPSS item. A marked drop in total IPSS was observed in all treatment arms following dosing, and there was a similar distribution of item-level IPSS responses at the three key trial visits (baseline, the landmark time point, and end-of-trial) in both the placebo arm and the pooled treatment arms. From Fig. 1, there was no apparent dose-response for the effect of degarelix on the IPSS.

**Table I:** Baseline Demographic and International Prostate Symptom Score (IPSS) Characteristics in Clinical Trial CS36

Variable	Placebo	Degarelix 10 mg	Degarelix 20 mg	Degarelix 30 mg
Number of patients	98	101	99	105
Age in years (median [range])	65.0 [50.0, 86.0]	65.0 [50.0, 81.0]	66.0 [52.0, 82.0]	65.0 [50.0, 87.0]
Body weight in kg (median [range])	86.4 [60.0, 128.0]	87.0 [54.1, 126.2]	85.0 [57.0, 141.2]	84.0 [55.0, 183.8]
Body mass index in kg/m/m (median [range])	28.5 [20.1, 40.2]	27.8 [18.9, 40.5]	27.7 [21.4, 38.9]	27.7 [19.8, 58.1]
Total IPSS (median [range])	18.0 [13.0, 33.0]	18.0 [11.0, 33.0]	19.0 [13.0, 33.0]	19.0 [13.0, 35.0]
IPSS storage subscore (median [range])	8.0 [3.0, 15.0]	8.0 [3.0, 15.0]	8.0 [4.0, 15.0]	8.0 [2.0, 15.0]
IPSS voiding subscore (median [range])	10.0 [4.0, 20.0]	11.0 [0.0, 20.0]	11.0 [3.0, 20.0]	11.0 [4.0, 20.0]
Quality of life score (median [range])	4.0 [2.0, 6.0]	4.0 [1.0, 6.0]	4.0 [2.0, 6.0]	4.0 [3.0, 6.0]
BPH Impact Index score (median [range])	7.0 [0.0, 13.0]	7.0 [0.0, 12.0]	7.0 [0.0, 12.0]	7.0 [0.0, 12.0]
Voided volume in mL (median [range])	175.5 [77.0, 466.0]	188.1 [125.0, 632.0]	185.0 [57.0, 505.0]	186.0 [106.4, 484.0]
Voiding time in s (median [range])	37.0 [19.0, 121.0]	40.0 [21.0, 128.0]	42.0 [15.0, 112.0]	39.0 [20.6, 344.5]
Post void residual volume in mL (median [range])	39.1 [0.0, 230.0]	50.5 [0.0, 246.6]	45.0 [0.0, 189.0]	56.3 [0.0, 999.0]
Average flow rate in mL/s (median [range])	5.0 [2.6, 10.4]	5.0 [2.6, 9.5]	5.3 [2.7, 10.6]	5.0 [2.3, 8.5]
Maximum urine flow in mL/s (median [range])	10.0 [4.6, 16.4]	10.0 [4.4, 19.2]	10.0 [5.4, 50.0]	9.9 [5.1, 16.0]
Flow time including time to maximum flow in s (median [range])	33.0 [18.0, 113.0]	36.0 [20.0, 120.0]	37.4 [13.0, 101.0]	37.0 [20.6, 100.4]
Total prostate volume in mL (median [range])	39.1 [16.8, 102.0]	38.4 [14.2, 128.0]	38.3 [17.0, 155.7]	36.1 [9.8, 135.9]
Prostate specific antigen in ng/mL (median [range])	2.0 [0.2, 9.6]	1.8 [0.1, 9.0]	2.3 [0.3, 9.6]	1.8 [0.3, 7.8]
Serum testosterone in ng/mL (median [range])	4.1 [1.0, 10.2]	4.3 [0.2, 13.6]	4.3 [2.0, 8.0]	4.3 [0.6, 12.2]
Region North America (*N*, %)	57 (58.2)	60 (59.4)	60 (60.6)	63 (60.0)
Region Europe (*N*, %)	41 (41.8)	41 (40.6)	39 (39.4)	42 (40.0)

### Item Response Theory Analysis

The unidimensional IRT model had high (> 0.6) item factor loadings except for the nocturia item, which had a modest factor loading value of 0.39, suggesting adequacy of the unidimensionality assumption. Factor analysis with two dimensions identified items relating to voiding (the emptying, intermittency, weak stream, and straining IPSS items) and storage (the frequency, urgency, and nocturia IPSS items) symptoms, respectively, as belonging to separate dimensions, informing the development of a bidimensional IRT model (item factor loading values are shown in Supplemental Table S1).

#### Unidimensional Item Characteristic Curve Estimation Model

**Fig. 2:**
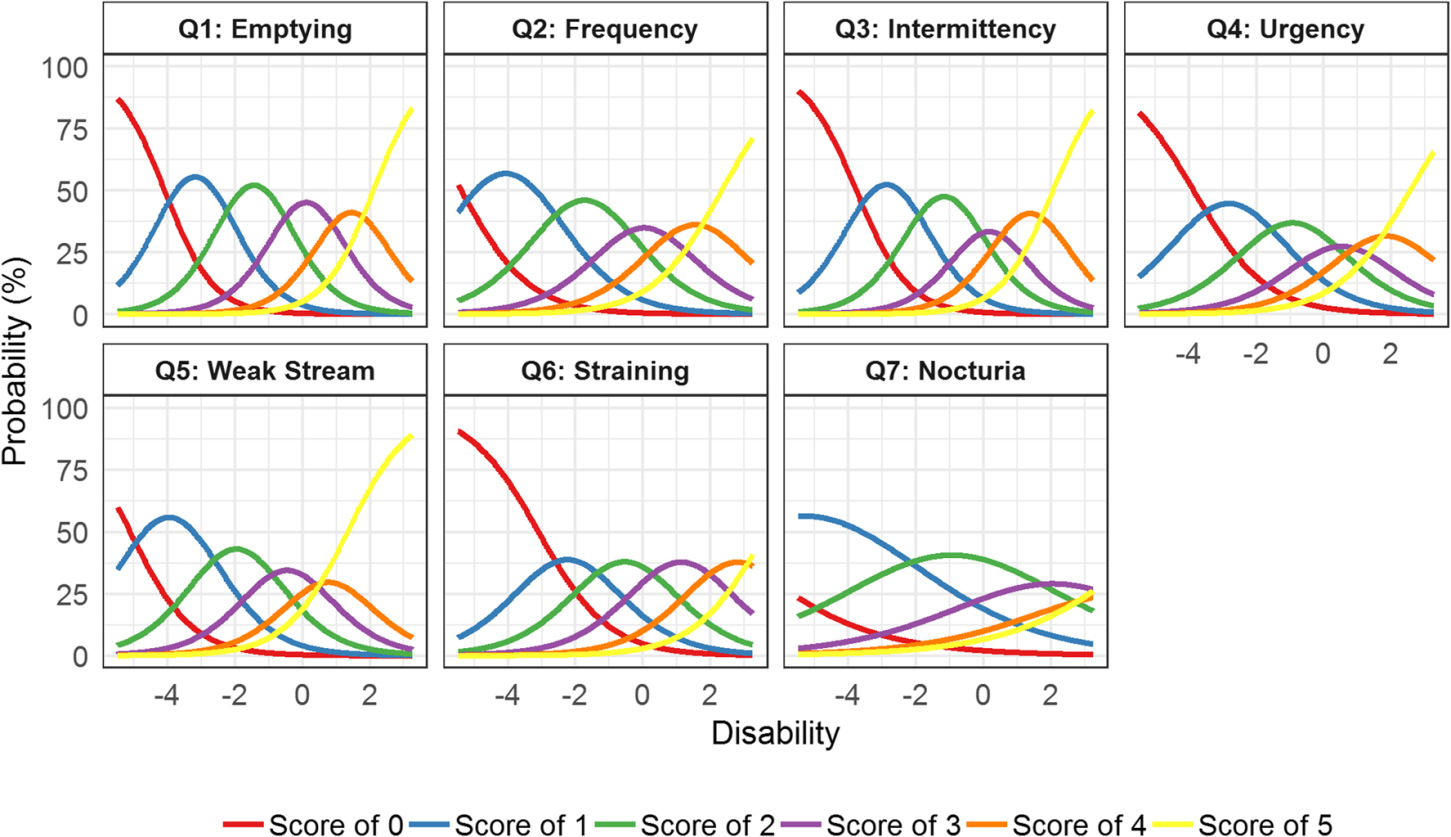
Item characteristic curves for each International Prostate Symptom Score item in the unidimensional item response theory model

In the unidimensional IRT ICC estimation model, 44 parameters (35 difficulty parameters, 7 discrimination parameters, and 2 post-baseline shift disability parameters) were estimated with low uncertainty in order to characterize the ICCs (Table II). The incomplete emptying IPSS item had the highest discrimination parameter value (1.38); i.e., it is more sensitive to changes in disability around the difficulty parameter of each score. The nocturia item had the lowest discrimination parameter value (0.49), indicating that a large increase in disability gives a relatively small increase in probability of increased score. The ICCs of each IPSS item are illustrated in Fig. 2 and show expected scores larger than zero for individuals with low disability (< − 4) for all items, most notably for the frequency, weak stream, and nocturia items. For the nocturia item, individuals with a low disability estimate are predominantly expected to score higher than 0, indicating that the vast majority of patients will answer that they get up to urinate at least once every night.

**Table II:** Item Characteristic Curve (ICC) Parameter Estimates in the (a) Unidimensional and (b) Bidimensional Item Response Theory (IRT) models

	a		b	
	Unidimensional model		Bidimensional model	
Parameter	Estimate	Relative standard error (%)	Estimate	Relative standard error (%)
IRT ICC parameters				
*a*_1_	1.38	7.0	1.6	7.6
*b*_1,1_	− 4.09	5.9	− 3.4	7.2
*b*_1,2_	1.82	7.4	1.56	8.1
*b*_1,3_	1.68	6.7	1.44	7.4
*b*_1,4_	1.41	6.8	1.2	7.6
*b*_1,5_	1.27	8.0	1.09	8.5
*a*_2_	0.98	7.0	1.4	8.5
*b*_2,1_	− 5.39	6.0	− 4.83	7.4
*b*_2,2_	2.64	7.5	2.24	8.3
*b*_2,3_	2.04	6.7	1.8	7.8
*b*_2,4_	1.49	7.1	1.3	8.2
*b*_2,5_	1.55	7.8	1.3	8.2
*a*_3_	1.29	7.7	1.68	8.2
*b*_3,1_	− 3.77	6.0	− 3.03	7.4
*b*_3,2_	1.8	7.4	1.48	8.0
*b*_3,3_	1.6	7.1	1.32	7.7
*b*_3,4_	1.08	7.5	0.88	8.0
*b*_3,5_	1.34	8.1	1.1	8.4
*a*_4_	0.92	6.7	1.16	8.0
*b*_4,1_	− 3.86	5.6	− 3.65	7.3
*b*_4,2_	2.09	6.8	1.88	8.1
*b*_4,3_	1.68	6.6	1.55	7.7
*b*_4,4_	1.22	7.2	1.12	8.0
*b*_4,5_	1.42	7.7	1.27	8.7
*a*_5_	1.09	7.2	1.36	7.7
*b*_5,1_	− 5.11	6.3	− 4.16	7.3
*b*_5,2_	2.31	7.8	1.9	8.3
*b*_5,3_	1.69	7.0	1.4	7.7
*b*_5,4_	1.32	7.1	1.09	7.7
*b*_5,5_	1.12	7.5	0.93	8.1
*a*_6_	0.95	7.8	1.25	8.2
*b*_6,1_	− 3.1	6.1	− 2.46	7.5
*b*_6,2_	1.72	7.7	1.38	8.2
*b*_6,3_	1.68	7.5	1.35	8.1
*b*_6,4_	1.67	9.8	1.34	8.3
*b*_6,5_	1.67	8.4	1.34	10.1
*a*_7_	0.49	8.4	0.601	8.5
*b*_7,1_	− 7.89	7.5	− 6.93	7.7
*b*_7,2_	5.19	8.7	4.4	8.5
*b*_7,3_	3.52	8.1	3.04	8.2
*b*_7,4_	2.44	8.9	2.09	8.9
*b*_7,5_	2.1	10.5	1.77	10.2
**Post-baseline shift parameters**				
Mean latent variable dimension 1	− 1.38	6.1	− 1.07	8.8
Variance latent variable dimension 1	2.22	6.4	1.61	7.3
Mean latent variable dimension 2	-	-	− 1.40	8.5
Variance latent variable dimension 2		–	2.4	7.4
Correlation between dimensions	-	-	69.1	3.6

*a*_*i*_ is the discrimination parameter for item *i*; *b*_*i,k*_ is the difficulty parameter for item *i* and category *k*. In the bidimensional model, dimension 1 (voiding) consists of items 1, 3, 5, and 6 while dimension 2 (storage) includes items 2, 4, and 7. At baseline, the latent variable(s) was fixed to *N*(0, 1) while the mean and variance of the latent variable(s) was estimated for post-baseline data (IDVIS approach)

Item #1: “Incomplete Emptying”; Item #2: “Frequency”; Item #3: “Intermittency”; Item #4: “Urgency”; Item #5: “Weak Stream”, Item #6: “Straining”, Item #7: “Nocturia”

**Fig. 3:**
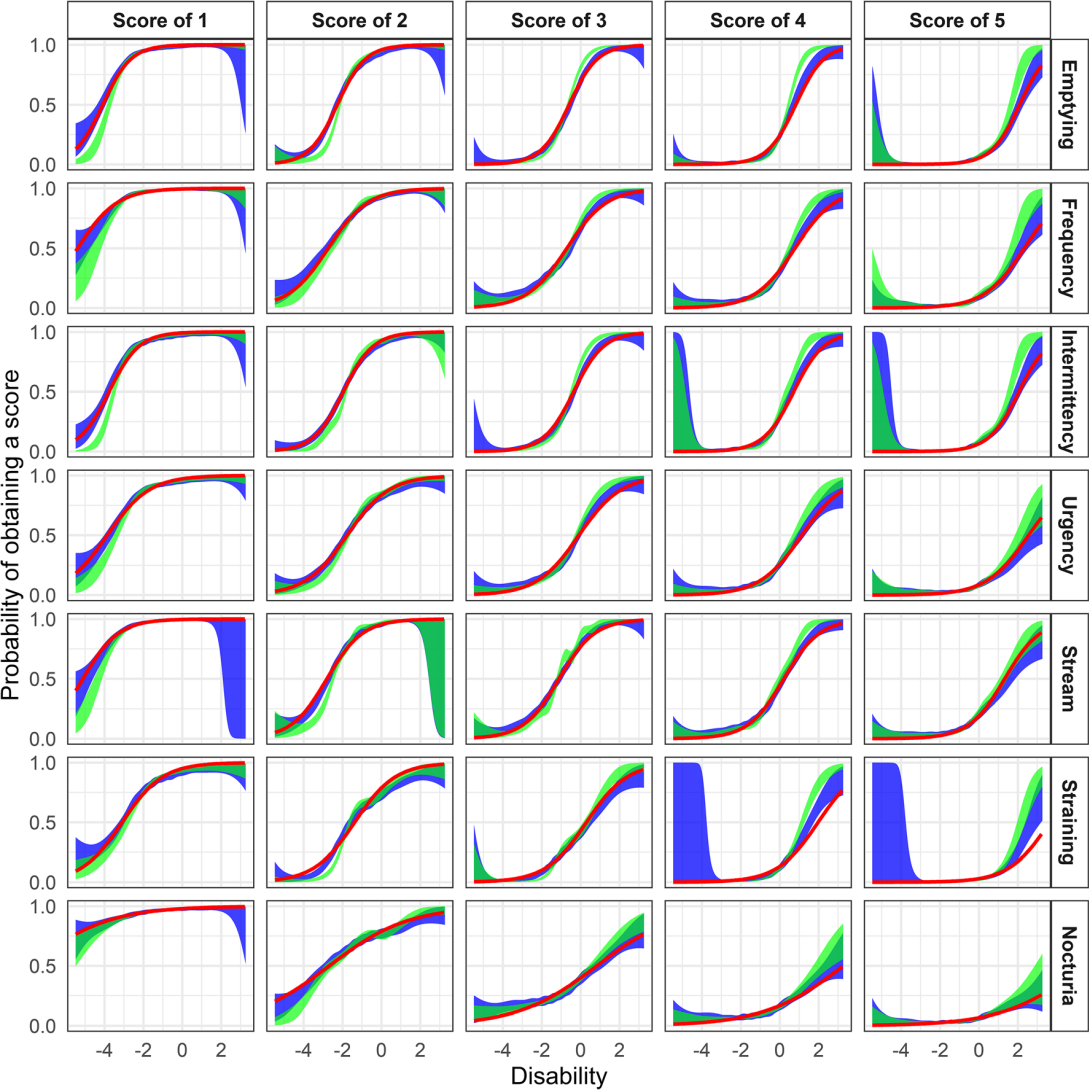
The International Prostate Symptom Score (IPSS) item characteristic curve fits in the unidimensional item response theory model for the cumulative probabilities (red lines) along with cross-validated cubic spline generalized additive model (GAM) smooth (green area) and *η* sampling-based cross-validated cubic spline GAM smooth using 200 samples (blue area)

Both the traditional cross-validated cubic spline GAM smooth and the sampling-based extension of the latter indicated that the estimated ICCs described the data adequately (Fig. 3). Better model agreement was observed with the sampling-based GAM smooth compared with the traditional method, although low typical *η*-shrinkage (SD-based) (9.6%) and low individual shrinkage variability (95% CI 9.6% to 9.9%, range 6.3% to 42.0%) was observed.

**Fig. 4:**
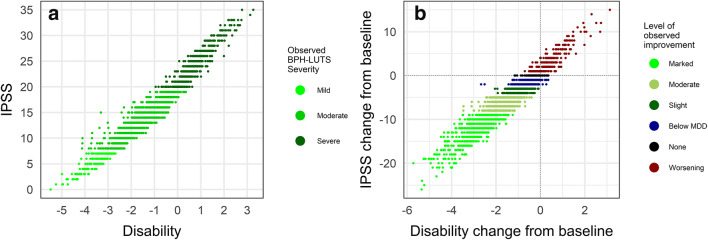
**a** Observed International Prostate Symptom Scores (IPSS) *vs.* item response theory disability estimates from the unidimensional item response theory model based on 3117 separate measurements from 403 patients over the 6-month trial period. **b** Observed change from baseline in International Prostate Symptom Scores (IPSS) *vs.* change from the baseline of item response theory disability from the unidimensional item response theory model in 403 patients over the 6-month trial period. MDD minimally detectable difference

Total IPSS spanning the entirety of the scale were observed in the CS36 data and high correlation (*r*^2^ = 0.95) with estimated IRT disability was observed (Fig. 4a). However, for a given summary IPSS value, there exists a wide range of underlying disability, most evident for moderate BPH-LUTS (8 ≤ IPSS ≤ 19). Moreover, Fig. 4b illustrates that the minimal detectable decrease (MDD) of three IPSS points (36,37) corresponds to a wide range of decreases in latent disability. In turn, there is a notable overlap between the latter disability improvements and those corresponding to observed improvements below the MDD (− 3 < ΔIPSS < 0), no observed change (ΔIPSS = 0), and to a small extent observed worsening (ΔIPSS > 0). Lastly, the threshold commonly used to determine clinical progression (ΔIPSS ≥ 4) (37–40) corresponds to no change or increases in underlying disability.

As shown in Table III, the most informative IPSS item was incomplete emptying (23.8% of total information), closely followed by intermittency (20.8% of total information). These items can determine patients’ disability more precisely relative to the other IPSS items. The nocturia item was found to contain the least information (3.4%), which is in line with this item having the lowest discrimination parameter value (Table II). Of note, the IPSS voiding items (incomplete emptying, intermittency, weak stream, and straining) combined carried 72% of the total information while IPSS storage items (frequency, urgency, and nocturia) combined only contained 28% of the total information. A visual representation of the Fisher information curves for each item is shown in Supplemental Fig. S2.

**Table III:** Fisher Information Content Ranking of International Prostate Symptom Score (IPSS) Items Based on the Unidimensional Item Response Theory Model

IPSS item	Item subscore category	% of total Fisher information	Cumulative % total
Q1: Incomplete Emptying	Voiding	23.8	23.8
Q3: Intermittency	Voiding	20.8	44.6
Q5: Weak Stream	Voiding	15.4	60
Q2: Frequency	Storage	13.1	73.1
Q6: Straining	Voiding	11.8	84.9
Q4: Urgency	Storage	11.6	96.5
Q7: Nocturia	Storage	3.4	99.9

#### Bidimensional Item Characteristic Curve Estimation Model

In the bidimensional IRT ICC estimation model, 47 parameters were estimated with low uncertainty (35 difficulty parameters, 7 discrimination parameters, two sets of post-baseline shift disability parameters, and a correlation term between latent variables) using Cholesky decomposition (to estimate the correlation between the latent variables fixed to 1). The bidimensional ICC estimation model had a 407.5 lower OFV than the unidimensional ICC estimation model, and its IRT parameter estimates and ICCs are presented in Table II and visually represented in Supplemental Figs. S3 and S4, respectively. Estimated ICCs adequately described the data as shown in Supplemental Figs. S5 and S6. Typical *η*-shrinkage was 10% (individual shrinkage 95% CI 9.8% to 10%, range 6.9% to 38.6%) and 13% (individual shrinkage 95% CI 13.6% to 13.8%, range 9.8% to 38.8%) in the voiding and storage dimension, respectively.

The residual correlation between items in the two respective developed IRT ICC estimation models is shown in Supplemental Figs. S7 and S8.

### Longitudinal Models

Three longitudinal models were developed: a total score model, a unidimensional IRT model, and a bidimensional IRT model. All three developed models adequately described the data as illustrated by VPCs (Supplemental Figs. S9, S10, S11, S12, and S13).

The time course of IPSS and latent disability in the summary score and unidimensional IRT model, respectively, were described according to

$$ I\mathrm{PSS}\ \mathrm{or}\ \mathrm{Disability}=\mathrm{Baseline}+\mathrm{Placebo}+\mathrm{Drug} $$

where Baseline is the estimated baseline, Drug is the offset degarelix treatment effect, and Placebo is the placebo effect described by

$$ \mathrm{Placebo}=\mathrm{Pmax}\left(1-{e}^{-\frac{\ln (2)}{\mathrm{Tprog}}\ast \mathrm{Time}}\right)+\mathrm{Drift}\ast \mathrm{Time} $$

where Pmax is the maximal placebo effect, Tprog is the half-life to reach Pmax, and Drift describes worsening or continued improvement.In the bidimensional IRT model, the placebo effect in each dimension was described using a Weibull function

$$ \mathrm{Placebo}=\mathrm{Pmax}\left(1-{e}^{-{\left(\frac{\ln (2)}{\mathrm{Tprog}}\ast \mathrm{Time}\right)}^{\mathrm{WEI}}}\right)+\mathrm{Drift}\ast \mathrm{Time} $$

where WEI is the Weibull exponent. Separate offset drug effects were estimated on each of the two latent variable scales.

Final longitudinal model parameter estimates for the total IPSS and unidimensional IRT model, along with their precision, are shown in Table IV. The lowest OFV and best goodness of fit were achieved by specifying log-normally distributed inter-individual variability (IIV) for Baseline_IPSS_ and Tprog_IPSS_ and normally distributed IIV for Pmax_IPSS_, and Drift_IPSS_. In longitudinal latent disability modeling, log-normal IIV was specified for Tprog_Disability_, while normal distributions were specified for Baseline_Disability_, Pmax_Disability_, and Drift_Disability_. The typical value of Drift was fixed to zero, and no significant changes in OFV were observed by doing so. The addition of IIV on Drug was not feasible in neither longitudinal IPSS nor latent disability modeling, as it yielded no significant OFV decrease and a variance close to zero, indicating that placebo and drug effect variability could not be distinguished in the current data. Incorporation of the offset drug effect into the total IPSS model, unidimensional IRT model, and bidimensional IRT model gave an OFV reduction of 22.1 (df = 1), 20.3 (df = 1), and 42.5 (df = 2), respectively, compared with the respective models without an estimated drug effect. No dose-response or exposure-response using AUC_0-∞_ as the exposure metric was observed on the IPSS and latent disability scale, respectively.

**Table IV:** Longitudinal model parameter estimates. IPSS: summary International Prostate Symptom Score, IRT: Item response theory. Relative standard errors were obtained in NONMEM

	IPSS model		Unidimensional IRT model	
Parameter	Value	Relative standard error (%)	Value	Relative standard error (%)
Baseline	19.6	1.7	0.0283	146.3
Pmax (maximal placebo response)	− 4.12	9.9	− 1.03	10.9
Tprog (placebo half-life)	15.3	18.8	12.3	20.5
Drug effect	− 1.98	19.2	− 0.542	20.3
Baseline Box-Cox shape	1.87	41.7	0.373	25.4
Drift Box-Cox shape	39.3	47.6	-	-
Covariates				
Baseline QoL on Pmax	0.208	13.2	-	-
Baseline BII on Baseline	0.0211	19.6	0.121	17.9
Baseline QoL on Baseline	0.0873	12.7	0.325	17.4
Region on Baseline	− 0.0803	26	− 0.338	24.1
Interindividual variability (IIV)				
IIV Baseline	13.7%	8.3	75.9%	7.7
IIV Pmax	121.7%	15.4	128.5%	15.4
IIV Drift	1.8%	19.4	0.7%	8.8
IIV Tprog	90.6%	12	52.4%	9.9
IIV Baseline-Pmax correlation	-	-	1.7%	
IIV Baseline-Drift correlation	-	-	9.2%	
IIV Pmax-Drift correlation	43.1%		34%	
Residual error				
Proportional residual error	10.9%	8.9		
Additive residual error	189.2%	6.7		

In the longitudinal the total IPSS and unidimensional IRT model, covariates were tested on the Base, Pmax, and Drug parameters. Significant covariates (*p* < 0.001) on Baseline in both models consisted of the baseline BII score, baseline QoL score, and study region, while baseline QoL score was included on Pmax_IPSS_ (Table IV). Due to the long runtime of the longitudinal full ICC model, covariates were identified using the longitudinal PSI-IPPSE approach and were subsequently incorporated into the full longitudinal ICC model. Re-estimation of the longitudinal parameters in the latter yielded an OFV decrease of approximately 130 points, and substantially better fit was observed in the VPCs of the item-level and summary-level IPSS (data not shown). Simultaneous re-estimation of ICCs and longitudinal parameters (estimates shown in Supplemental Table S2) yielded an OFV decrease of 11 points compared with the fixed ICC longitudinal unidimensional IRT model. This was deemed insignificant, and hence, the longitudinal unidimensional IRT model with fixed ICCs and estimated longitudinal parameters was kept as the final model. In the latter, covariate relationships found to be significant using the PSI-IPPSE method underwent an additional backward elimination step (< 0.001) to confirm their significance. All covariates remained statistically significant in the full model. Lastly, Box-Cox transformation of the Baseline and Drift IIV distributions in both models resulted in significant drops in OFV. However, in longitudinal unidimensional IRT modeling, the Box-Cox shape parameter had a high relative standard error (> 400%) and was therefore ultimately not included as part of the final model.

During longitudinal bidimensional IRT modeling, high correlation (≥ 96%) was observed between the Tprog IIV and Pmax IIV components for each dimension, which affected model stability. These IIV parameters were hence collapsed into a single common parameter across the two dimensions. The typical value of the Weibull exponent was also estimated to be the same in both dimensions due to model stability. As per the unidimensional IRT model, longitudinal parameters were re-estimated in the final longitudinal bidimensional IRT model. The final model minimized successfully and its parameter estimates are shown in Table V. It was not possible to obtain parameter precision estimates, include covariates, or simultaneously estimate ICCs and longitudinal parameters due to convergence and stability issues. The final bidimensional longitudinal IRT model adequately described both summary and item level data (Supplemental Figs. S12 and S13, respectively).

**Table V:** Parameter estimates for the longitudinal bidimensional item response theory model

Parameter	Value
Baseline_V_ (voiding scale)	− 0.0251
Baseline_S_ (storage scale)	− 0.0667
Pmax_V_ (maximal placebo response voiding scale)	− 0.75
Pmax_S_ (maximal placebo response storage scale)	− 0.845
Tprog_V_ (placebo half-life voiding scale)	12.9
Tprog_S_ (placebo half-life storage scale)	13.4
Weibull shape parameter (common for both scales)	1.53
Drug effect voiding scale	− 0.488
Drug effect storage scale	− 0.749
Interindividual variability (IIV)	
IIV Baseline_v_ (voiding scale)	97.3%
IIV Baseline_S_ (storage scale)	128.8%
IIV Baseline_v_-Baseline_S_ correlation	26%
IIV Pmax (common for both scales)	145.6%
IIV Tprog (common for both scales)	61.1%
IIV Drift (common for both scales)	0.6%
IIV Pmax-Drift correlation	40%

### Power of Testing and Model-Based Methods

**Fig. 5:**
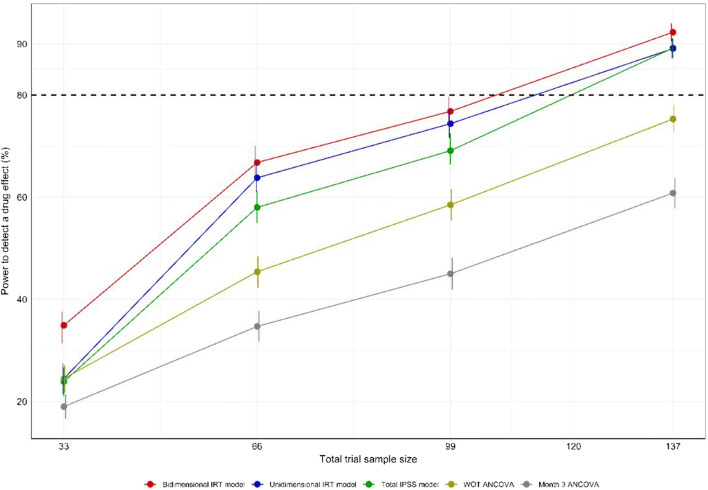
Power curves for the pharmacometric models obtained using a type I error corrected stochastic simulation and estimation procedure. One thousand simulated data sets from the bidimensional item response theory model at sample sizes of 33, 66, 99, and 137 patients were used for model estimation with the respective full (with a drug effect parameter) and reduced (without a drug effect parameter) models. Vertical lines indicate the 95% confidence interval for the calculated power estimates

The bidimensional IRT model was used as the simulation model in the SSE procedure as it provided a lower AIC value (59,086.3) compared with the unidimensional IRT model (AIC value of 61,622.6). The resulting power curves are shown in Fig. 5. The pharmacometric models all provided considerably higher power to detect a drug effect compared with the cross-sectional ANCOVA as well as the WOT ANCOVA. The unidimensional IRT model yielded slightly higher power (approximately *N* = 113 to reach 80% power) compared with the total IPSS model (approximately *N* = 120 to reach 80% power). An additional SSE procedure confirmed this finding, using the unidimensional IRT model as simulation model (data not shown). The bidimensional IRT model provided the highest power to detect a drug effect, allowing for a total trial sample of approximately *N* = 106 to reach 80% power compared with the total IPSS and unidimensional IRT models. The type 1 error of each model under each sample size and empirically derived OFV cut-off in the SSE procedure is presented in the Supplemental Table S3. Only model runs that minimized successfully were used in the calculation of power (on average ~ 80% of full-reduced bidimensional model pairs and ~ 90% of unidimensional and total IPSS model pairs, respectively).

## DISCUSSION

### Item Response Theory Analysis

The current paper presents the first reported IRT analyses of the IPSS and longitudinal pharmacometric IRT model within BPH-LUTS. Both a unidimensional and a bidimensional IPSS IRT model were developed based on factor analyses, the latter further confirming previous findings (41,42).

In the unidimensional IRT model, the vast majority of the total information content was contained in IPSS voiding items and this finding is supported by a principal component analysis showing total IPSS being predicted by improvement in voiding symptoms rather than storage symptoms (43). Subscore analysis, i.e., distinguishing treatment effects on the IPSS voiding and storage subscores in addition to the total IPSS, is routinely performed as a secondary statistical analysis of clinical trials within BPH-LUTS, although its clinical meaningfulness has not been established (42,44,45). The current results suggest that the IPSS voiding subscore is more sensitive in assessing a patient’s BPH-LUTS in comparison with the storage subscore and may therefore also be better suited for detecting symptomatic drug effects. It is however to be noted that the most favorable signal-to-noise ratio will be obtained by regarding all available data and acknowledging the information contribution of individual items as opposed to considering the composite (sub)score(s), as exampled by pharmacometric IRT in Parkinson’s disease (15).

The incomplete emptying item was found to be the most informative. This item has previously been found to be associated with worsening of both voiding and storage symptoms (46). Incomplete emptying had the highest discrimination parameter value (1.38) in the unidimensional IRT model; however, compared with other reported unidimensional IRT analyses in different therapeutic areas, this is relatively low (e.g., the highest discrimination parameter value was 3.35 in the ADAS-cog IRT analysis (9) and 3.5 in the EDSS IRT analysis (12)). This may indicate that BPH-LUTS is a diffuse and heterogeneous disease, and consequently, IPSS items have difficulty in discriminating between different levels of disability.

The nocturia item was found to be the least informative, and several reports in the literature support this. Firstly, the item may not be sufficiently specific to BPH-LUTS; the primary cause of adult nocturnal polyuria has been attributed to the decline in nocturnal secretion of antidiuretic hormone due to aging (47,48) as opposed to being a direct consequence of BPH. The nocturia item was also the least specific in Japanese men with BPH and a similar explanation was proposed (49). Secondly, nocturia may be unspecific to urologic conditions in general. Significant correlation between IPSS nocturia and items 5 and 6 describing nocturia in the 8-item overactive bladder questionnaire (OAB-8) has been established (50); an IRT analysis of the OAB-8 in both men and women showed the two items describing nocturia to have the relatively lowest discrimination parameter values (51) (ratio to the highest discrimination parameter estimate was 0.35, 0.40, and 0.42 for IPSS nocturia, OAB-8 item 5, and OAB-8 item 6, respectively). It should be emphasized that nocturia and urgency symptoms appear to be the most bothersome symptoms to patients suffering from LUTS (52,53). Lower information content does not entail that the corresponding symptom is not bothersome from a patient perspective; it indicates that the frequency of observed scores varies less across patients with highly different disease severity compared with other items. The item is therefore less sensitive in assessing the overall condition and less useful for distinguishing between patients. The bother of each BPH-LUTS symptom is expected to vary between patients, yet this is not captured by the IPSS; this diagnostic limitation (54) is addressed by other questionnaires, e.g., the Danish Prostate Symptom Score (55) and the International Continence Society Questionnaire Male LUTS questionnaire (56).

Based on comparison between IRT disability and total IPSS, the MDD of IPSS ≤ − 3 for classifying patients as experiencing clinically significant improvement (36,37) and IPSS ≥ 4 for determining clinical progression of BPH-LUTS (37–40) is supported. However, seeing that there is extensive overlap between changes in latent disability at the observed MDD and below it (decreases lower than three total IPSS points and to a certain extent increases in total IPSS), using only the change in total IPSS to evaluate response may overlook many patients that benefit from treatment. The same reasoning applies to patients that experience worsening of their symptoms.

Discussion regarding the developed sampling-based GAM smooth methodology for evaluating ICCs is presented in the Supplemental Discussion.

### Longitudinal Modeling

In both the longitudinal total IPSS and IRT models, a model describing treatment as present or absent best described treatment effect although three different drug doses (10 mg, 20 mg, and 30 mg) were included in the analyzed trial. Lack of observed dose-response and exposure-response relationships may be explained by the narrow dose range studied in the current trial. Including at least four active doses spanning an at least 10-fold range has previously been emphasized to characterize dose-exposure-response adequately (57). In the current trial, the width of the dose range was restricted due to the expectation of an increase in the incidence of prolonged testosterone suppression at higher doses of degarelix. Further discussion regarding longitudinal modeling and covariate analysis results are presented in the Supplemental Discussion.

The longitudinal bidimensional IRT model allowed for estimation of a differential drug effect on voiding and storage IPSS symptoms, while preserving item-level information. This approach may be more in line with the different effects of therapy on the primary pathophysiologies behind voiding and storage symptoms (58,59). Limitations of the pharmacometric bidimensional model included lack of longitudinal parameter precision estimates and inability to include covariates. This can be attributed to the increased model complexity due to presence of several latent variables, and other longitudinal pharmacometric multidimensional IRT models have reported similar issues (13,14). More advanced and computationally intensive methods for assessing parameter uncertainty (e.g., a non-parametric bootstrap) may be used to obtain parameter precision, but were beyond the scope of the current work. Item- and summary-level VPCs were therefore the primary basis for concluding adequate model fit and predictive performance. If longitudinal model stability and covariate identification are of primary interest, the longitudinal unidimensional IRT model may be a better-suited alternative. The unidimensional approach may also be advantageous for more straightforward translation between changes in the summary IPSS and IRT-estimated disability. From a psychometric standpoint, both the unidimensional and bidimensional IPSS IRT approaches are valid (41).

### Power

The longitudinal model-based analyses showed considerably higher power to detect a drug effect compared with the cross-sectional ANCOVA using only data from the visit 3 months post-dose. The higher power of longitudinal pharmacometric modeling compared with cross-sectional testing is not a novel finding and has previously been reported in several other therapeutic areas (9–11), yet comparison with a WOT estimand-based test has to our knowledge not been presented previously. These findings are discussed further in the Supplemental Discussion.

A modest increase in power to detect a drug effect was observed by the use of the unidimensional IRT modeling compared with the total IPSS model, and this finding was unexpected given that other longitudinal IRT applications have shown greater increases in power compared with longitudinal summary score modeling (9,15). Studies have shown that the larger the number of items in a questionnaire, the higher the power of IRT (60,61), and this may explain the similar power between the summary IPSS model and the unidimensional IRT model in the current study compared with analyses of questionnaires with a higher number of items. Furthermore, the heterogeneity in the item discrimination parameter values has been shown to affect the power of IRT compared with summary score modeling (62). For instance, for the 8-item Expanded Disability Status Scale (EDSS) in multiple sclerosis, pharmacometric IRT analysis showed a larger power increase compared with summary score modeling (63) than in the current study, which may be explained by the higher variability between discrimination parameter estimates of EDSS items (66% CV) compared with IPSS items (29% CV) (12). In the current work, the bidimensional pharmacometric IRT model was used for simulation of data on which the power to detect a drug effect was estimated for the unidimensional IRT and total IPSS models, respectively. A sensitivity analysis specifying the unidimensional IRT model as the simulation model was performed and confirmed the currently reported power difference between the pharmacometric unidimensional IRT model and the total IPSS model (data not shown).

A higher power to detect a drug effect was observed with the longitudinal bidimensional IRT model compared with the unidimensional IRT model. This may be due to the differences in ICCs and disability scale of the multidimensional model compared with the unidimensional model, which, in turn, give a more precise discernment of the drug effect. Given a questionnaire where multidimensionality is substantiated, we hypothesize that the difference in power to detect a drug effect may increase compared with a unidimensional IRT model as the correlation between latent variables decreases, as this would gradually increase the difference in ICCs and disability scale. This is the first investigation of the impact of IRT dimensionality on the power to detect a drug effect and hence warrants further investigation. For example, the original application of pharmacometric IRT based on the ADAS-cog scale (9) investigated the power of a unidimensional IRT model; based on findings suggesting that the ADAS-cog is multidimensional (64), it may also be of interest to assess the power of a multidimensional pharmacometric ADAS-cog IRT model.

A limitation of the current as well as previous pharmacometric IRT studies (9,15,63) was that simulation model bias was present in the power calculations: the pharmacometric IRT model used for simulation of data was also used to estimate power and may therefore have favored the pharmacometric IRT approaches. Other approaches, such as developing longitudinal ordered categorical models for each item and simulating data from these, were considered. However, it is not clear whether the IPSS ICCs would be preserved or require re-estimation based on simulated data by doing so and whether meaningful comparison with previously reported reductions in sample size would be feasible.

The current findings may serve to more precisely assess patients’ underlying BPH-LUTS by utilizing the available item-level IPSS responses instead of considering only the sum of these scores. Furthermore, they may inform more efficient clinical development of BPH-LUTS treatments, although the gain in power to detect a drug effect was found to be lower compared with previously reported applications with different scales describing different neurological conditions (9,15,63). IRT focuses on quantifying the information of questionnaires in specific patient populations; since the modeled data spanned the entire range of total IPSS (i.e., from the lowest to the highest possible disease severity), the presented results may be extended to the analysis of the IPSS in other clinical trials including similar patients with moderate-to-severe BPH-LUTS, regardless of treatment and its effect size.

5The current study emphasizes the importance of quantifying the increase in power to detect a drug effect with pharmacometric IRT modeling when applied to different measurement scales, as it may differ to a great extent depending on the internal characteristics of the latter. Knowledge regarding the size of the increase in the power to detect a drug effect may be primordial in informing a drug developer’s decision to implement the more complex IRT methodology. For completeness, it is to be noted that pharmacometric modeling of longitudinal data is not the current standard for detecting drug effects in clinical trials. Further research regarding, e.g., its general alignment with traditional statistical analyses, the adequacy of its underlying assumptions, its type I error control, and its pre-specification (65–67), is needed before it may be regarded as the primary analysis method and thereby dictate the sample size of clinical trials.

The IRT methodology may be implemented in all clinical trials where composite scores are used to assess treatment efficacy, i.e., from proof-of-concept phase II to confirmatory phase III trials. However, the shift from using “observed total score” to “underlying disease” as the estimand summary measure (32) may represent a substantial paradigm shift and may therefore require framework developments supervised by regulators. An example could be the development of standardized item banks based on a large number of item-level patient responses from many trials. This would inform precise ICCs and thereby allow for precise and, most importantly, consistent estimation of latent disability across different clinical trials. The merit and practical utility of IRT in increasing the efficiency of clinical development programs appear to already be recognized within the US Food and Drug Administration (68).

## CONCLUSION

Pharmacometric models were developed based on item-level and summary-level IPSS, respectively, to describe the time course of underlying disability and total IPSS in patients with moderate-to-severe BPH-LUTS in a clinical trial setting. IRT analysis revealed that voiding IPSS items combined contained the majority of the information content, which may have implications for the analysis of IPSS subscores. The unidimensional IRT model showed slightly higher power to detect a drug effect compared with the composite score model, while the bidimensional IRT model further increased the power. Taking the multidimensional nature of the IPSS into account in a pharmacometric IRT framework may hence allow for more precise quantification of drug effects and optimization of statistical power.

## Electronic Supplementary Material

ESM 1 (PDF 1353 kb)
