# Supplementary material for: Item Response Theory Modeling of the International Prostate Symptom Score in Patients with Lower Urinary Tract Symptoms Associated with Benign Prostatic Hyperplasia
Source: AAPS J. 2020 Aug 27;22(5):115. doi: 10.1208/s12248-020-00500-w (PMC7452927; doi:10.1208/s12248-020-00500-w)
Supplement: Supplementary file 1 — (PDF 1353 kb) [file 12248_2020_500_MOESM1_ESM.pdf]

Contents

1. **Supplemental Figure S1**: Item-level International Prostate Symptom Score distribution 2
2. **Supplemental Figure S2**: Fisher Information curves 3
3. **Supplemental Figures S3 and S4**: Bidimensional Item Response Theory Item Characteristic Curves 4
4. **Supplemental Figure S5 and S6**: Sampling-based GAM smooths Bidimensional Item Response Theory Model 5
5. **Supplemental Figure S7**: Residual correlation in the unidimensional item response theory model 7
6. **Supplemental Figure S8**: Residual correlation in the bidimensional item response theory model 8
7. **Supplemental Figure S9**: Longitudinal total score model visual predictive check 9
8. **Supplemental Figure S10**: Longitudinal unidimensional item response theory model summary-level visual predictive check 10
9. **Supplemental Figure S11**: Longitudinal unidimensional item response theory model item-level visual predictive checks 11
10. **Supplemenal Figure S12**: Longitudinal bidimensional item response theory model summary-level visual predictive checks 15
11. **Supplemenal Figure S13**: Longitudinal bidimensional item response theory model item-level visual predictive checks 16
12. **Supplemental Table S1**: Confirmatory Factor Analysis - Item factor loadings 20
13. **Supplemental Table S2**: Parameter estimates of simultaneous longitudinal unidimensional item response theory model 21
14. **Supplemental Table S3**: Stochastic simulation and estimation type I error 23
15. **Supplemental Discussion** 24

1. **Supplemental Figure S1**: Item-level International Prostate Symptom Score distribution

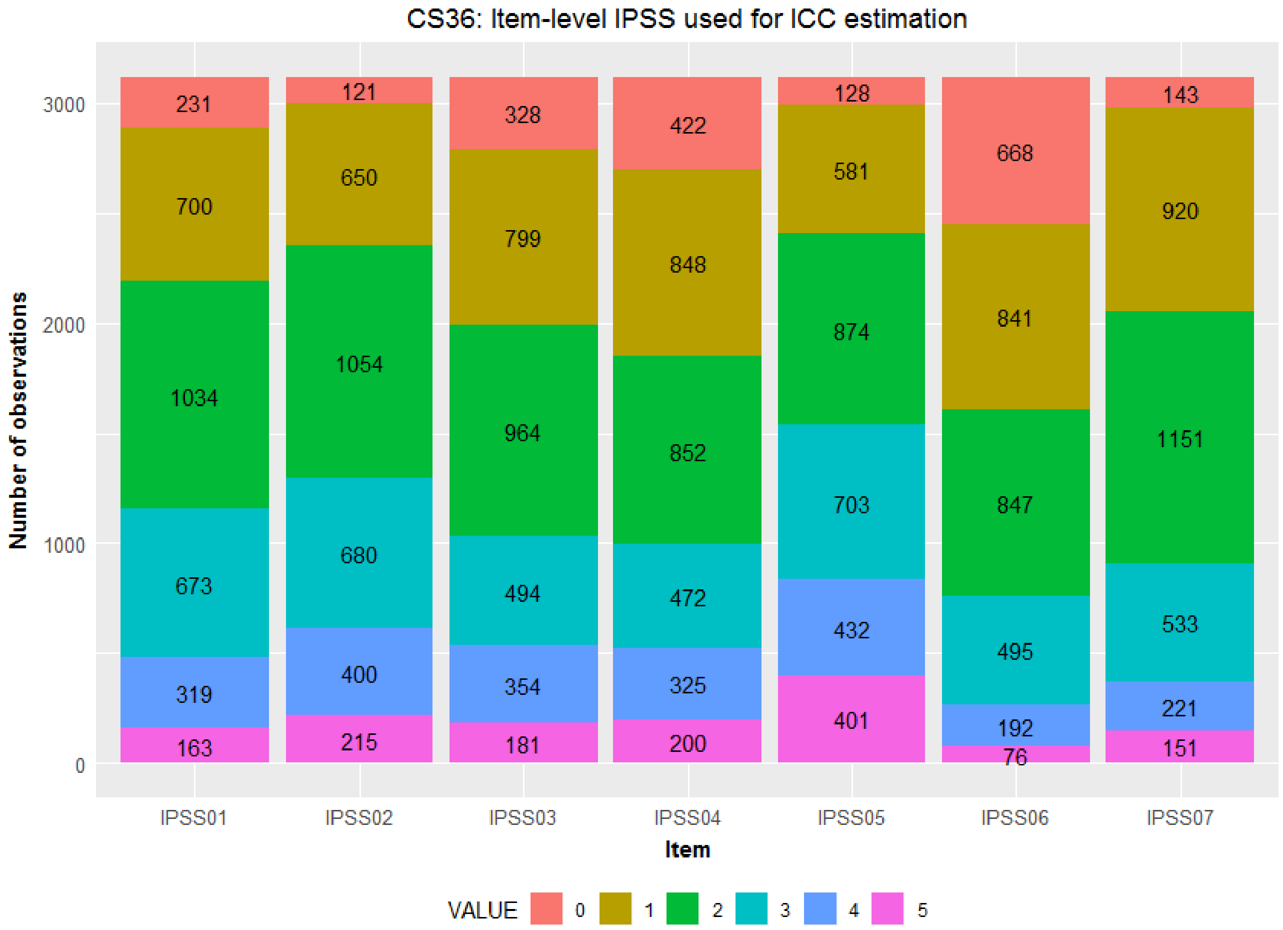

Supplemental Figure S1 – Response distribution for each of the seven International Prostate Symptom Score items in the CS36 data set. IPSS01: Item 1 "Incomplete Emptying"; IPSS02: Item 2 "Frequency"; IPSS03: Item 3 "Intermittency"; IPSS04: Item 4 "Urgency"; IPSS05: Item 5 "Weak Stream"; IPSS06: Item 6 "Straining"; IPSS07: Item 7 "Nocturia".

2. **Supplemental Figure S2**: Fisher Information curves

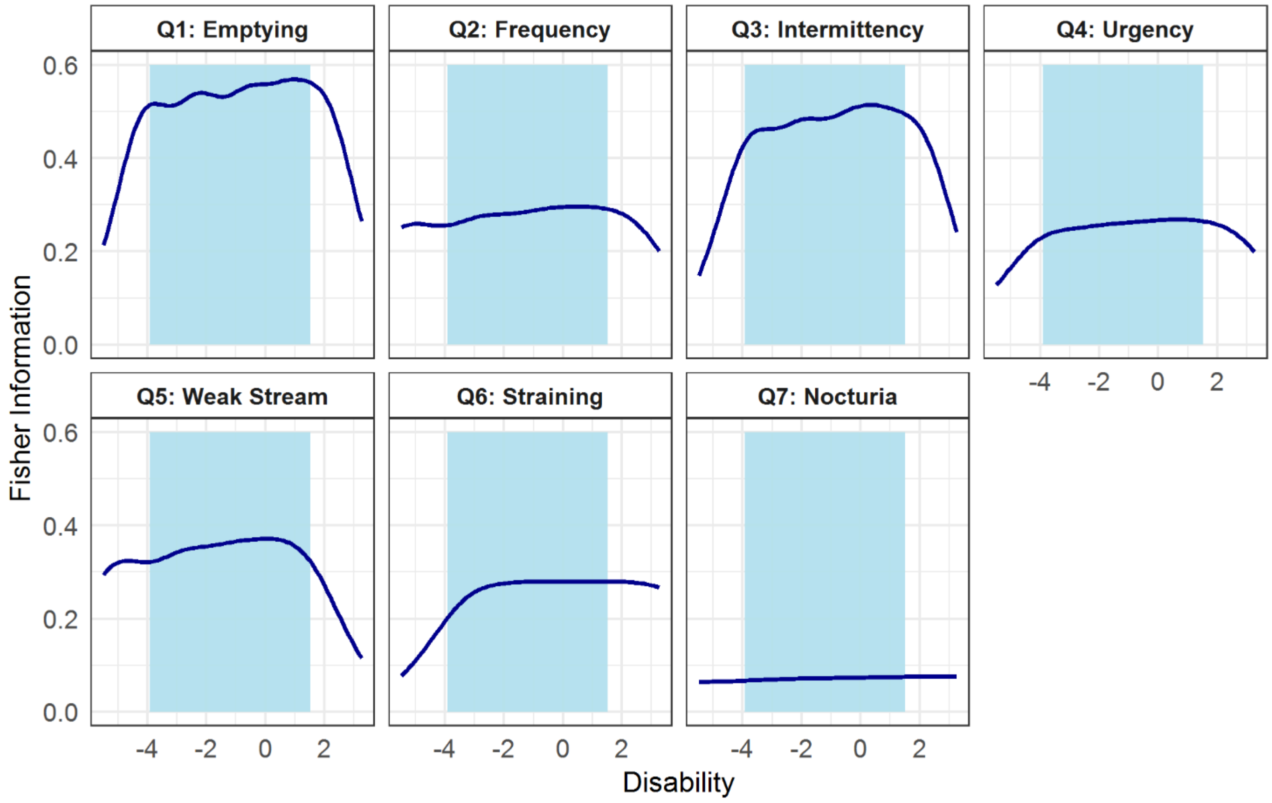

Supplemental Figure S2 – Fisher Information Content for International Prostate Symptom Score items versus Item Response Theory disability from the unidimensional item response theory model. Shaded areas indicate the disability range for 95% of the study population.

3. **Supplemental Figures S3 and S4**: Bidimensional Item Response Theory Item Characteristic Curves

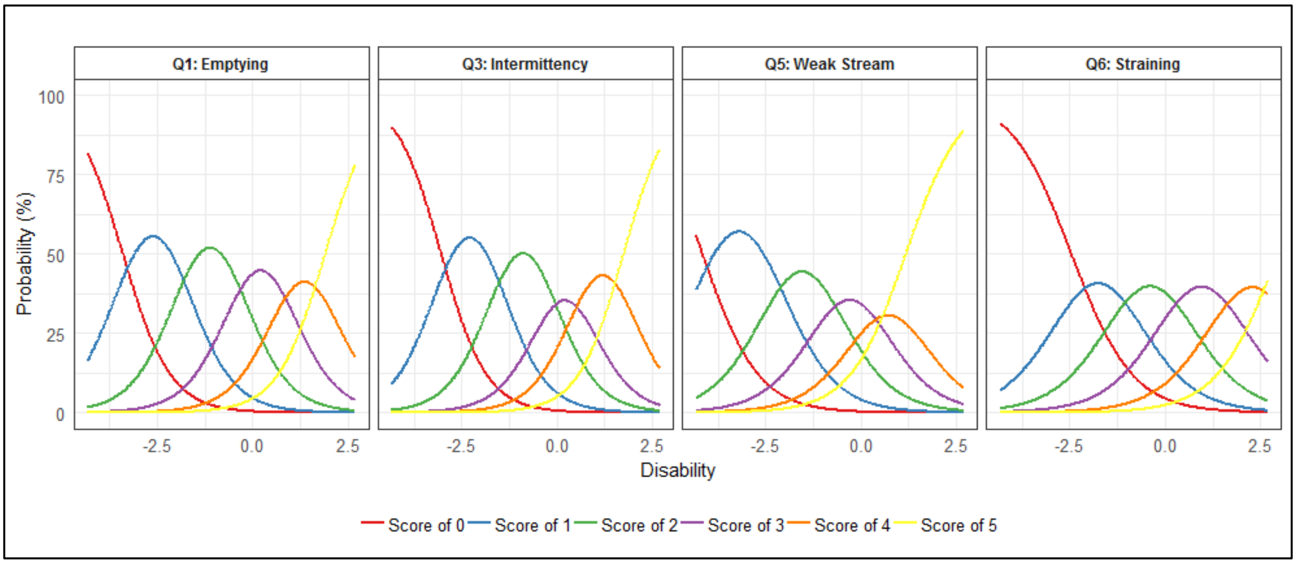

Supplemental Figure S3 – Item characteristic curves (ICCs) for International Prostate Symptom Score items related to voiding based on the bidimensional IRT ICC estimation model.

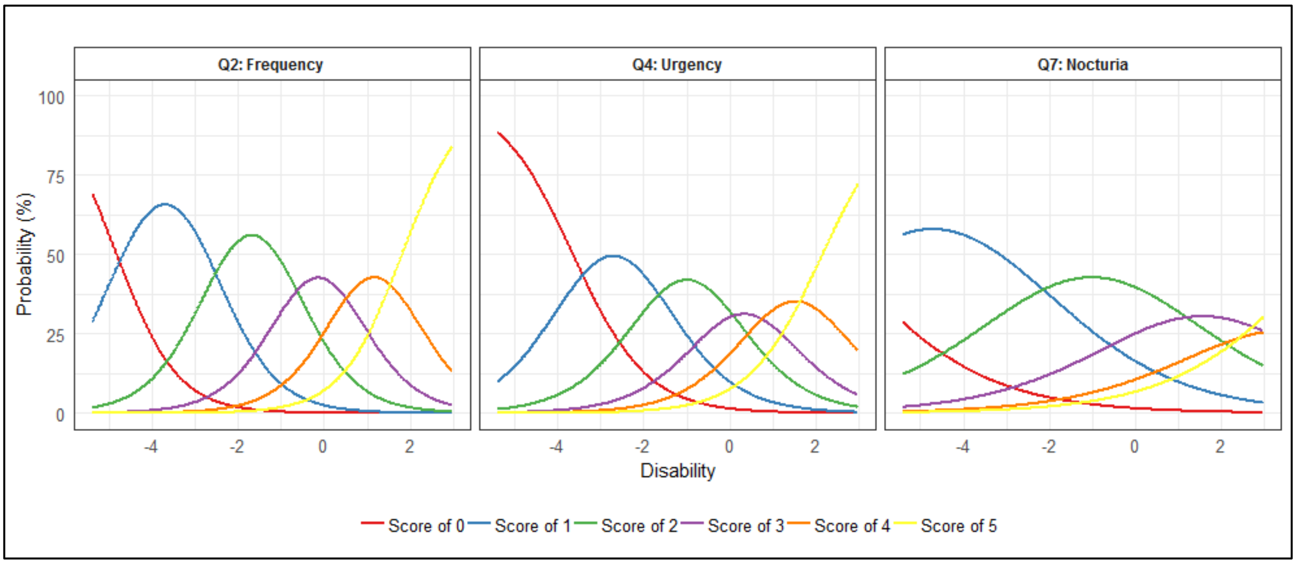

Supplemental Figure S4 – Item characteristic curves (ICCs) for International Prostate Symptom Score items related to storage in the bidimensional item response theory ICC estimation model.

4. **Supplemental Figure S5 and S6**: Sampling-based GAM smooths Bidimensional Item Response Theory Model

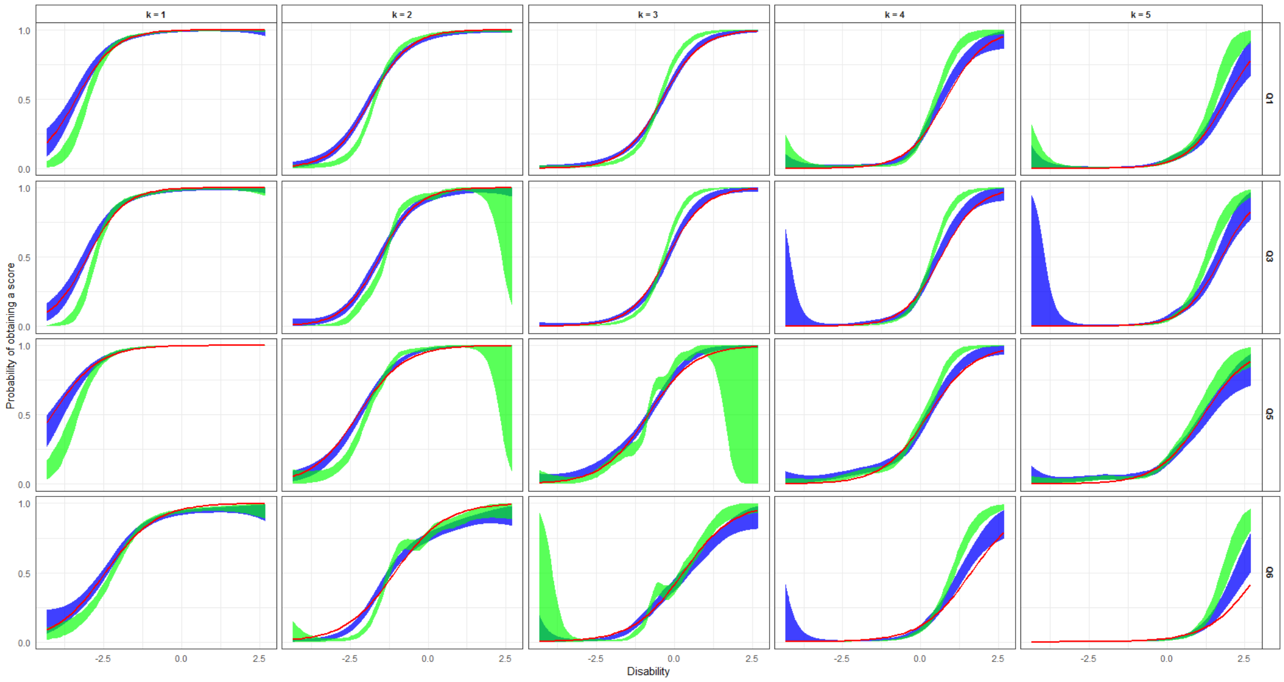

Supplemental Figure S5 – International Prostate Symptom Score (IPSS) voiding item characteristic curve fits in the bidimensional item response theory model for the cumulative probabilities (red lines) along with cross-validated cubic spline generalized additive model (GAM) smooth (green area) and η sampling-based cross-validated cubic spline GAM smooth using 200 samples (blue area). η-shrinkage was 10% on the standard deviation scale. *k* is the score. Q1: IPSS item 1 “Incomplete Emptying”, Q3: IPSS Item 3 “Intermittency”, Q5: IPSS Item 5 “Weak Stream”, Q6: IPSS Item 6 “Straining”.

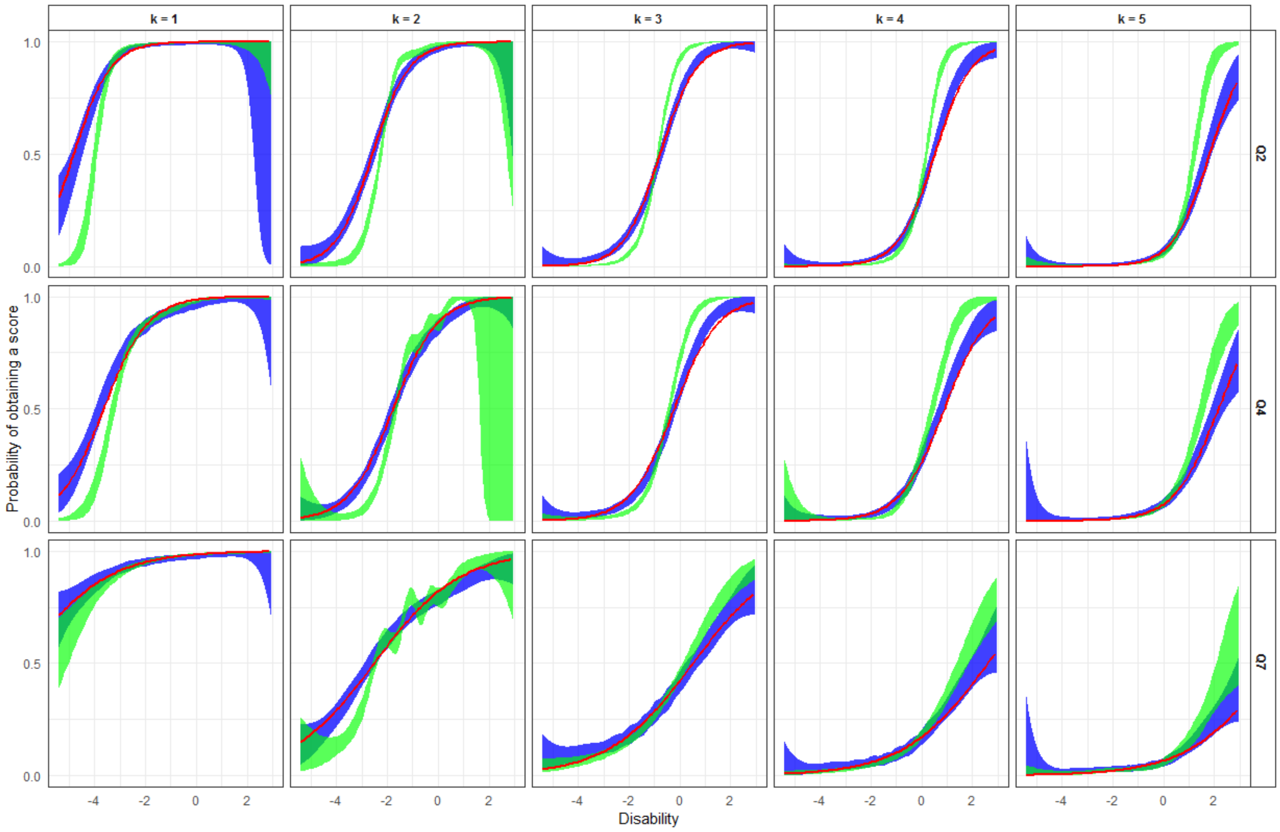

Supplemental Figure S6 – International Prostate Symptom Score (IPSS) storage item characteristic curve fits in the bidimensional item response theory model for the cumulative probabilities (red lines) along with cross-validated cubic spline generalized additive model (GAM) smooth (green area) and η sampling-based cross-validated cubic spline GAM smooth using 200 samples (blue area). η-shrinkage was 10% on the standard deviation scale. *k* is the score. Q2: IPSS Item 2 “Frequency”, Q4: IPSS Item 4 “Urgency”, Q7: IPSS Item 7 “Nocturia”.

5. **Supplemental Figure S7**: Residual correlation in the unidimensional item response theory model

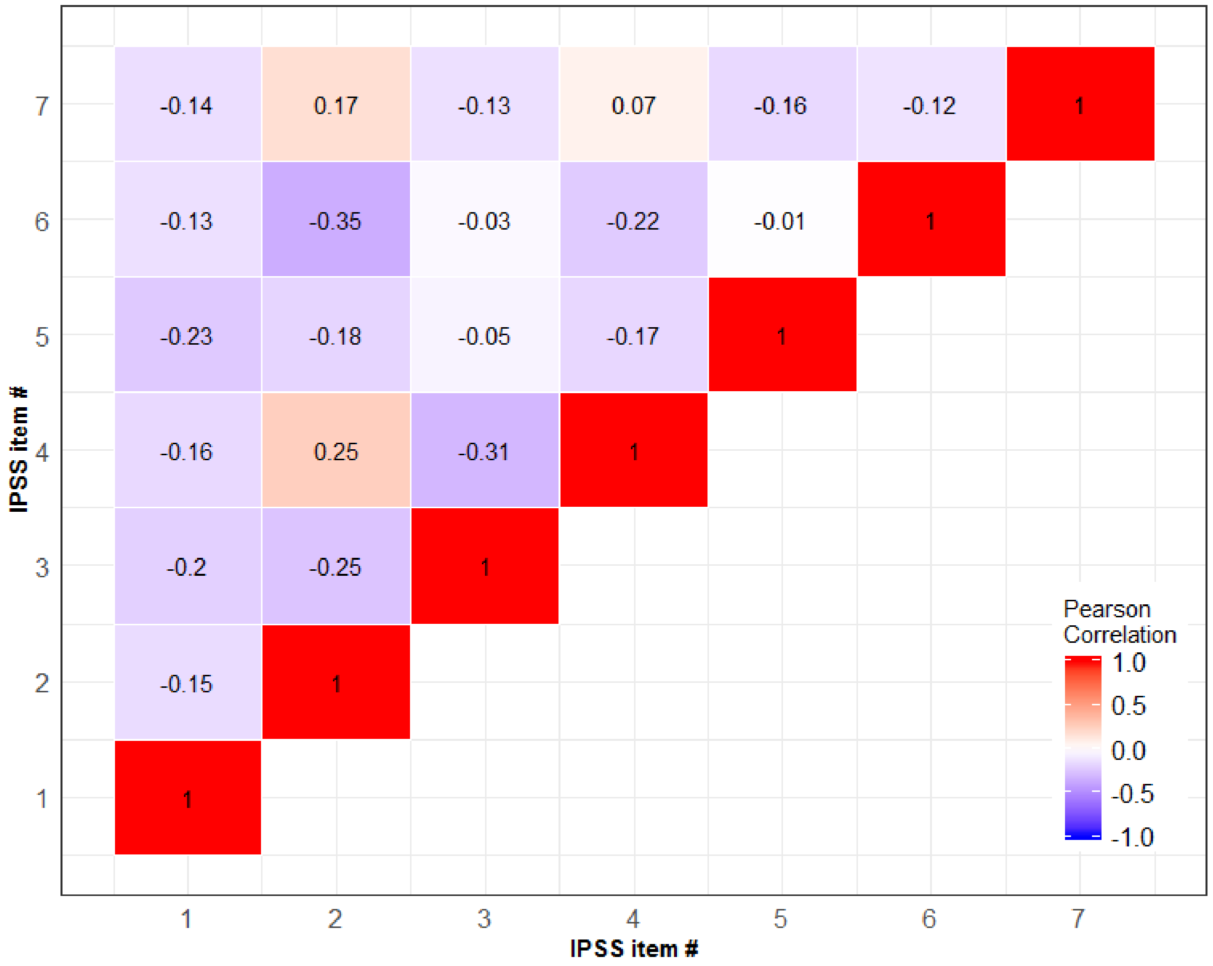

Supplemental Figure S7 – Residual correlation between item responses in the unidimensional item response theory item characteristic curve estimation model. Item #1: "Incomplete Emptying"; Item #2: "Frequency"; Item #3: "Intermittency"; Item #4: "Urgency"; Item #5: "Weak Stream", Item #6: "Straining", Item #7: "Nocturia".

6. **Supplemental Figure S8**: Residual correlation in the bidimensional item response theory model

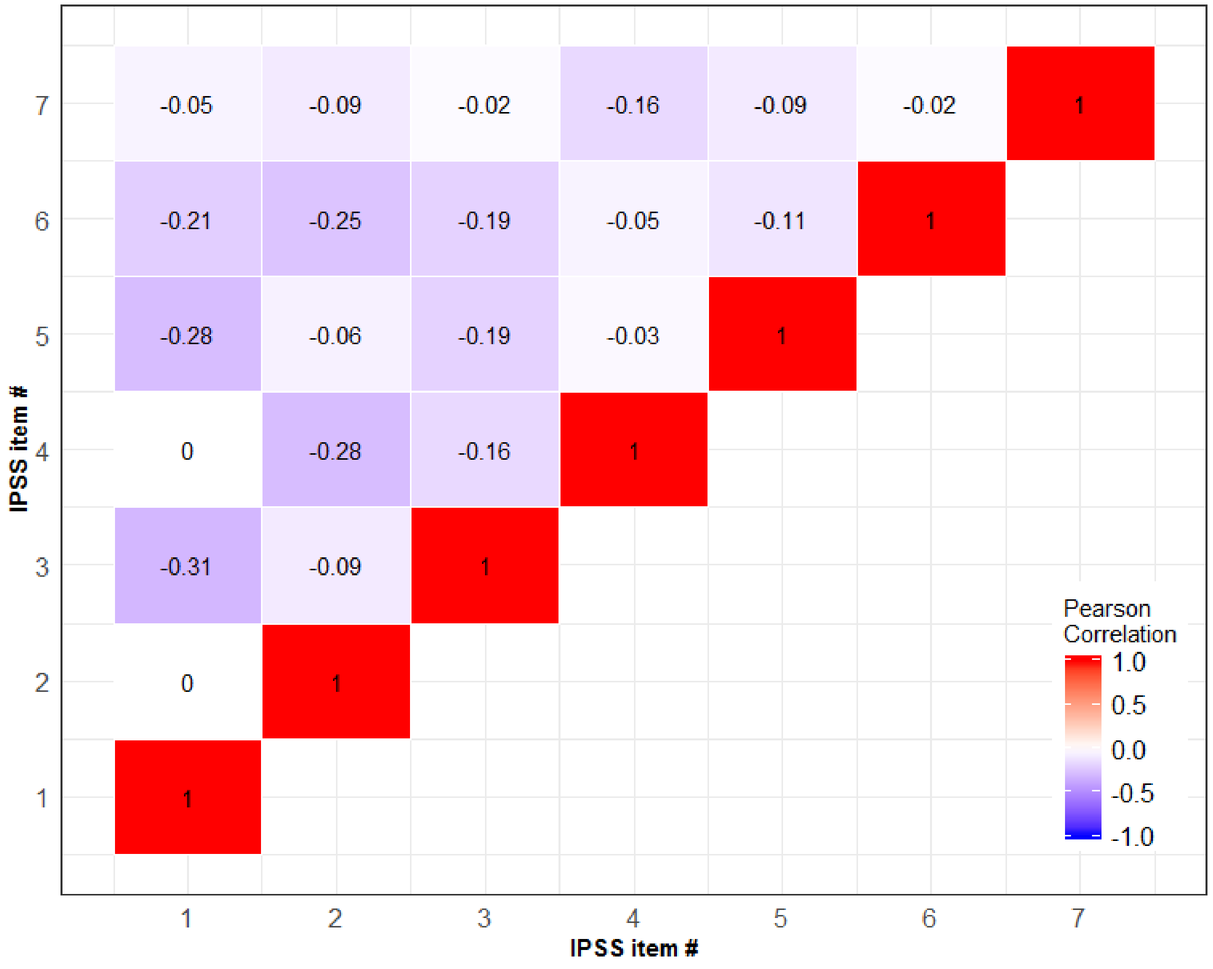

Supplemental Figure S8 – Residual correlation between item responses in the bidimensional IRT model, with separate latent variables for voiding (items 1, 3, 5 and 6) and storage (items 2, 4, and 7) symptoms. Item #1: “Incomplete Emptying”; Item #2: “Frequency”; Item #3: “Intermittency”; Item #4: “Urgency”; Item #5: “Weak Stream”, Item #6: “Straining”, Item #7: “Nocturia”.

7. **Supplemental Figure S9**: Longitudinal total score model visual predictive check

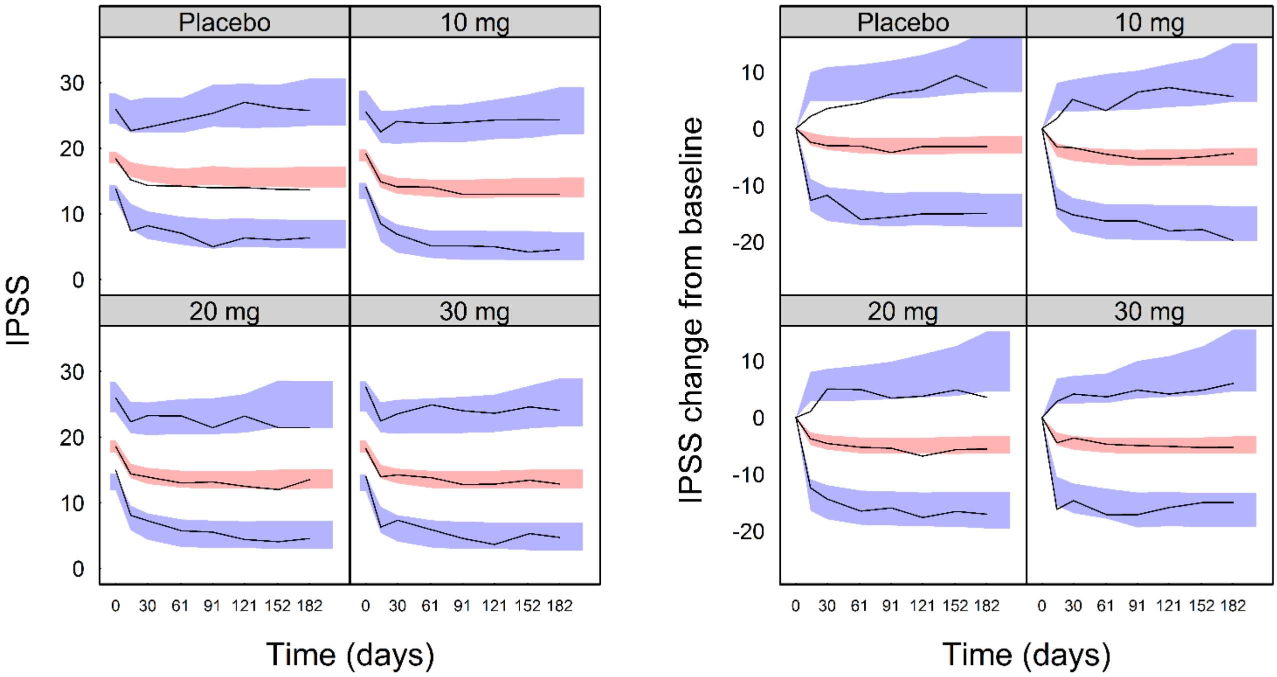

Supplemental Figure S9 –Visual predictive check of the longitudinal summary International Prostate Symptom Scores (IPSS) model stratified by treatment arm comparing the median, 2.5th, and 97.5th percentiles of the observed data with the corresponding percentiles for simulated data displayed as 95% confidence intervals. Treatment effect was modeled as absent (placebo arm) or present (10 mg, 20 mg, and 30 mg degarelix arms). 200 simulated data sets were used.

8. **Supplemental Figure S10**: Longitudinal unidimensional item response theory model summary-level visual predictive check

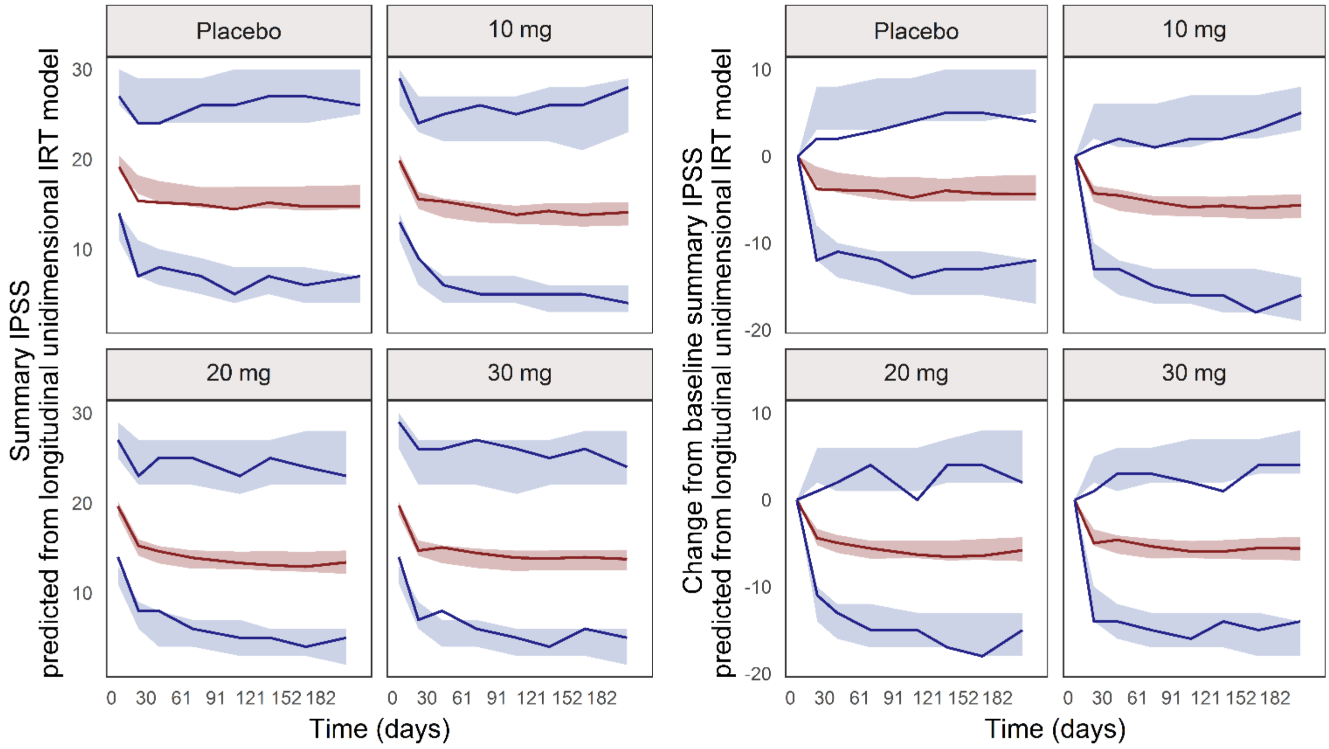

Supplemental Figure S10 – Visual predictive check for the unidimensional longitudinal IRT model stratified by treatment arm comparing the median, 2.5th, and 97.5th percentiles of the observed data with the corresponding percentiles for simulated data displayed as 95% confidence intervals. Treatment effect was modeled as absent (placebo arm) or present (10 mg, 20 mg, and 30 mg degarelix arms).

9. **Supplemental Figure S11**: Longitudinal unidimensional item response theory model item-level visual predictive checks

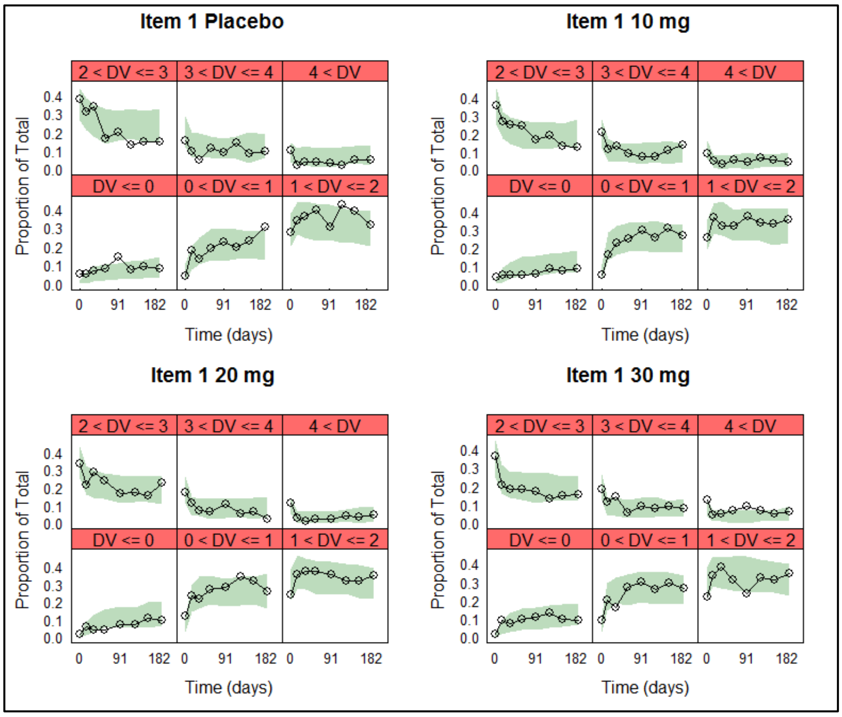

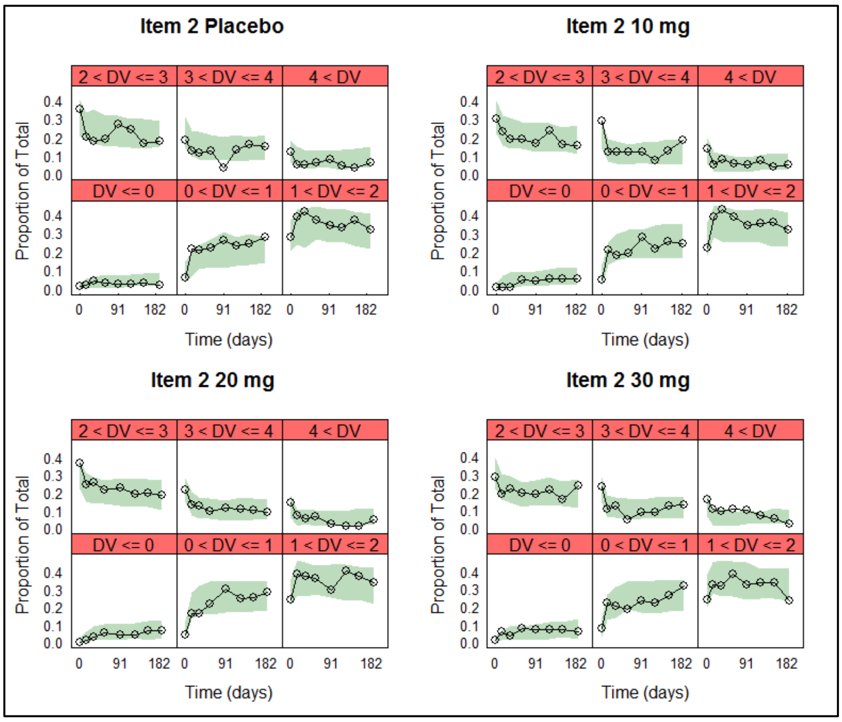

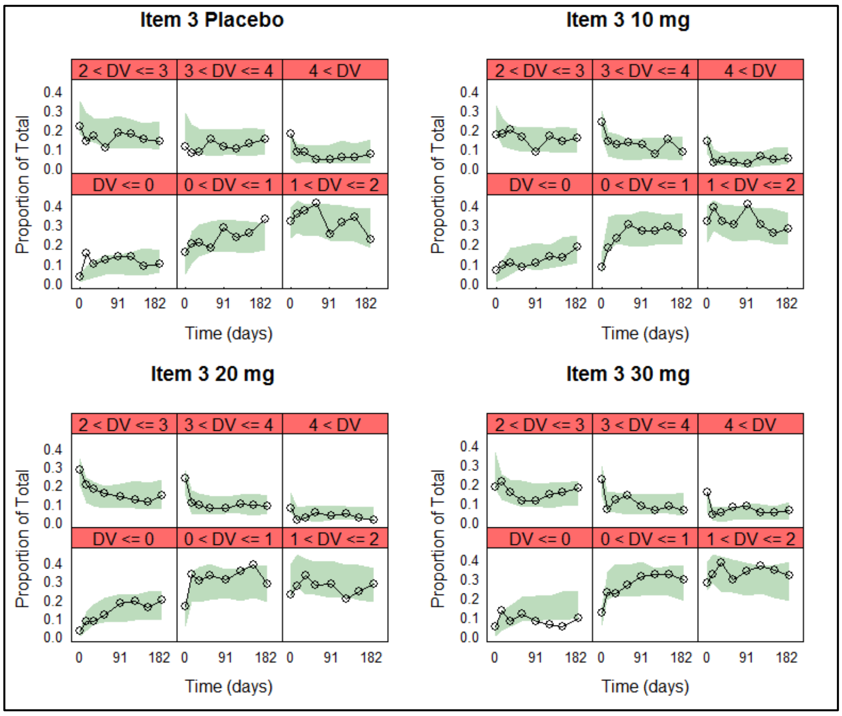

Item 3 Placebo
Item 3 10 mg
Item 3 20 mg
Item 3 30 mg
2 < DV <= 3
3 < DV <= 4
4 < DV
DV <= 0
0 < DV <= 1
1 < DV <= 2
Proportion of Total
Time (days)

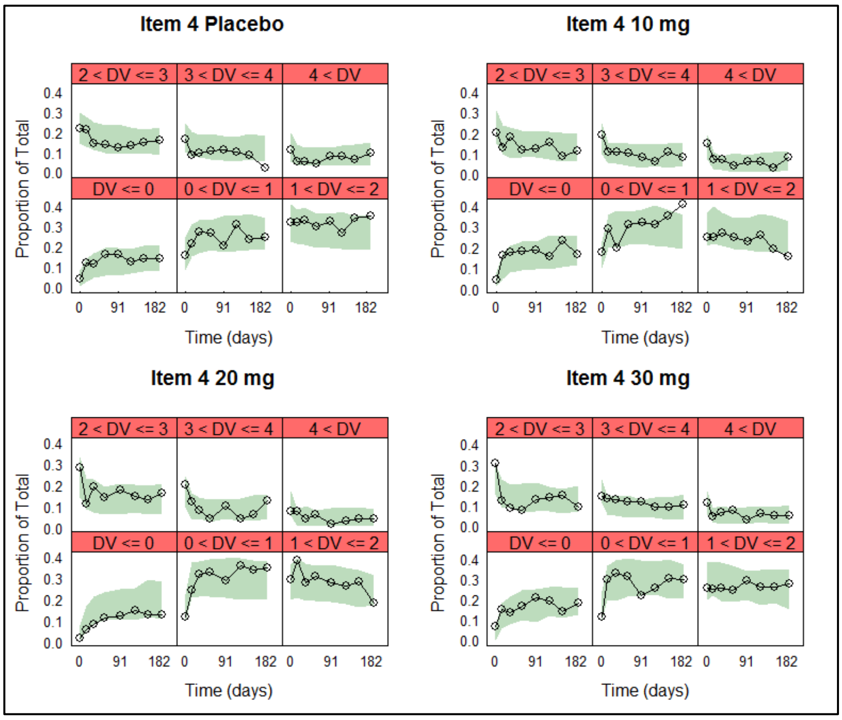

Item 4 Placebo
Item 4 10 mg
Item 4 20 mg
Item 4 30 mg
2 < DV <= 3
3 < DV <= 4
4 < DV
DV <= 0
0 < DV <= 1
1 < DV <= 2
Proportion of Total
Time (days)

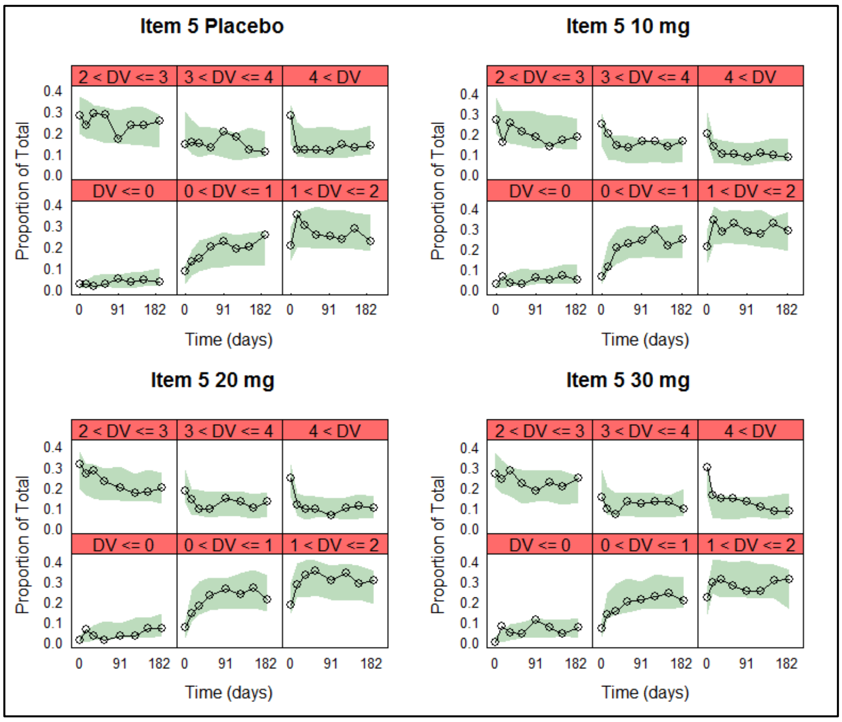

Item 5 Placebo
Item 5 10 mg
Item 5 20 mg
Item 5 30 mg
2 < DV <= 3
3 < DV <= 4
4 < DV
DV <= 0
0 < DV <= 1
1 < DV <= 2
Proportion of Total
0.0 0.1 0.2 0.3 0.4
0 91 182
Time (days)

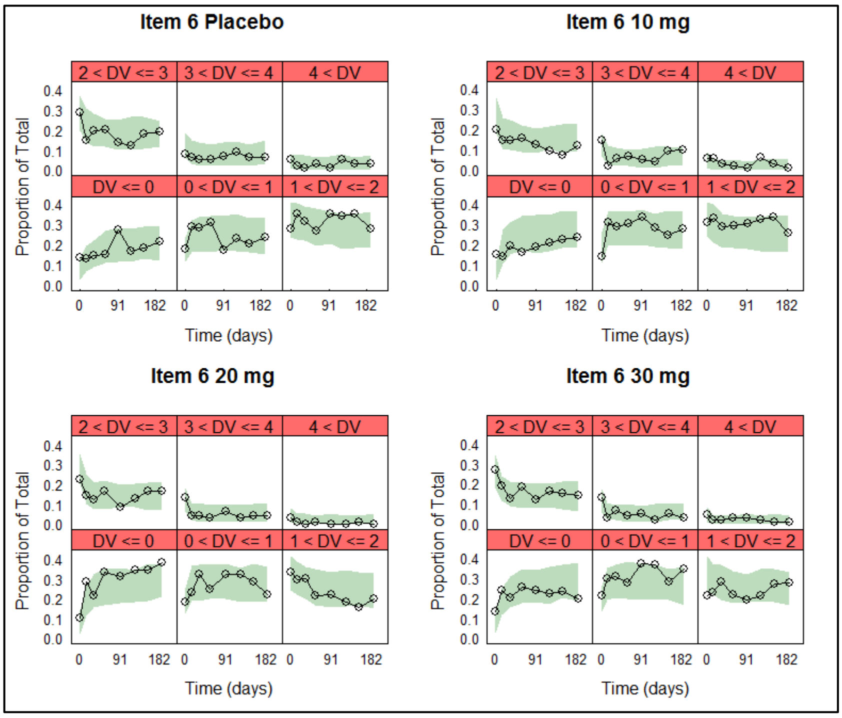

Item 6 Placebo
Item 6 10 mg
Item 6 20 mg
Item 6 30 mg
2 < DV <= 3
3 < DV <= 4
4 < DV
DV <= 0
0 < DV <= 1
1 < DV <= 2
Proportion of Total
0.0 0.1 0.2 0.3 0.4
0 91 182
Time (days)

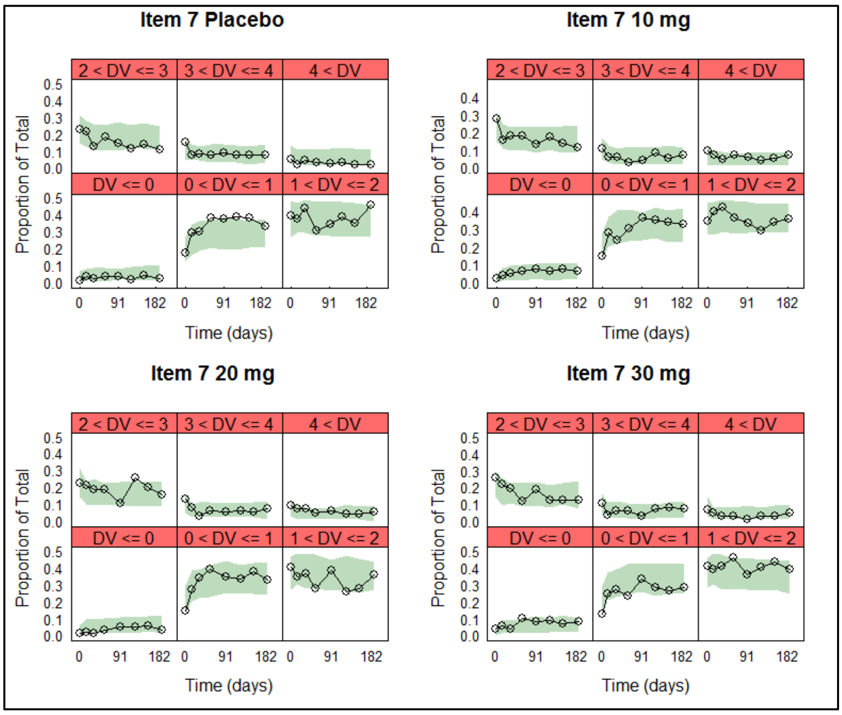

Supplemental Figure S11 – Item-level visual predictive checks in the unidimensional item response theory IPSS model stratified by treatment arm. The observed frequency of each score over time is shown as points and the shaded areas indicate the 95% confidence intervals of the frequencies of each score in 200 simulated datasets. DV: dependent variable, i.e., observed score.

10. **Supplemenal Figure S12**: Longitudinal bidimensional item response theory model summary-level visual predictive checks

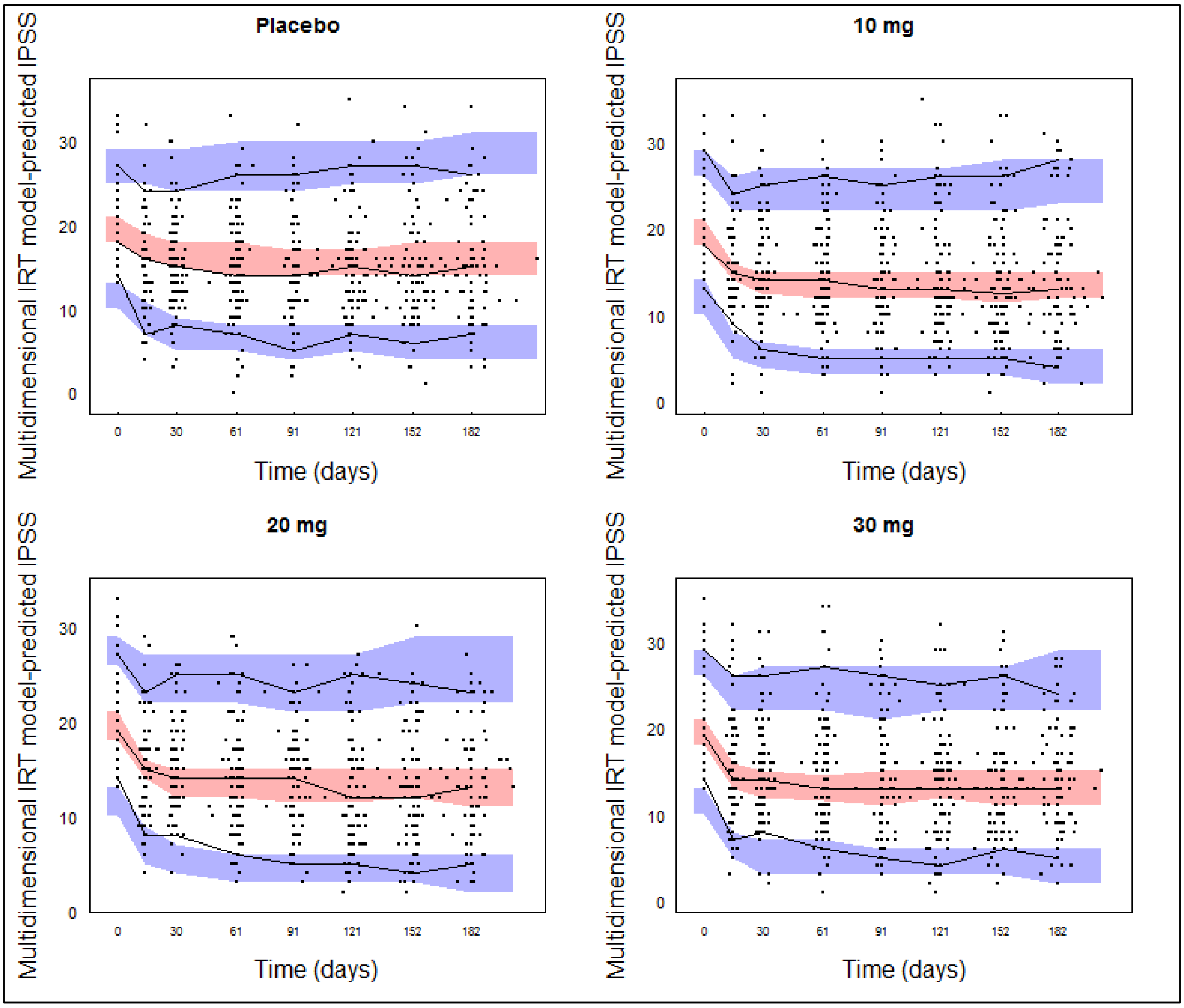

Supplemental Figure S12 – Summary-level visual predictive checks in the bidimensional item response theory IPSS model stratified by treatment arm comparing the median, 2.5th, and 97.5th percentiles of the observed data with the corresponding percentiles for simulated data displayed as 95% confidence intervals. Treatment effect was modeled as absent (placebo arm) or present (10 mg, 20 mg, and 30 mg degarelix arms). IPSS: International Prostate Symptom Score.

11. **Supplemenal Figure S13**: Longitudinal bidimensional item response theory model item-level visual predictive checks

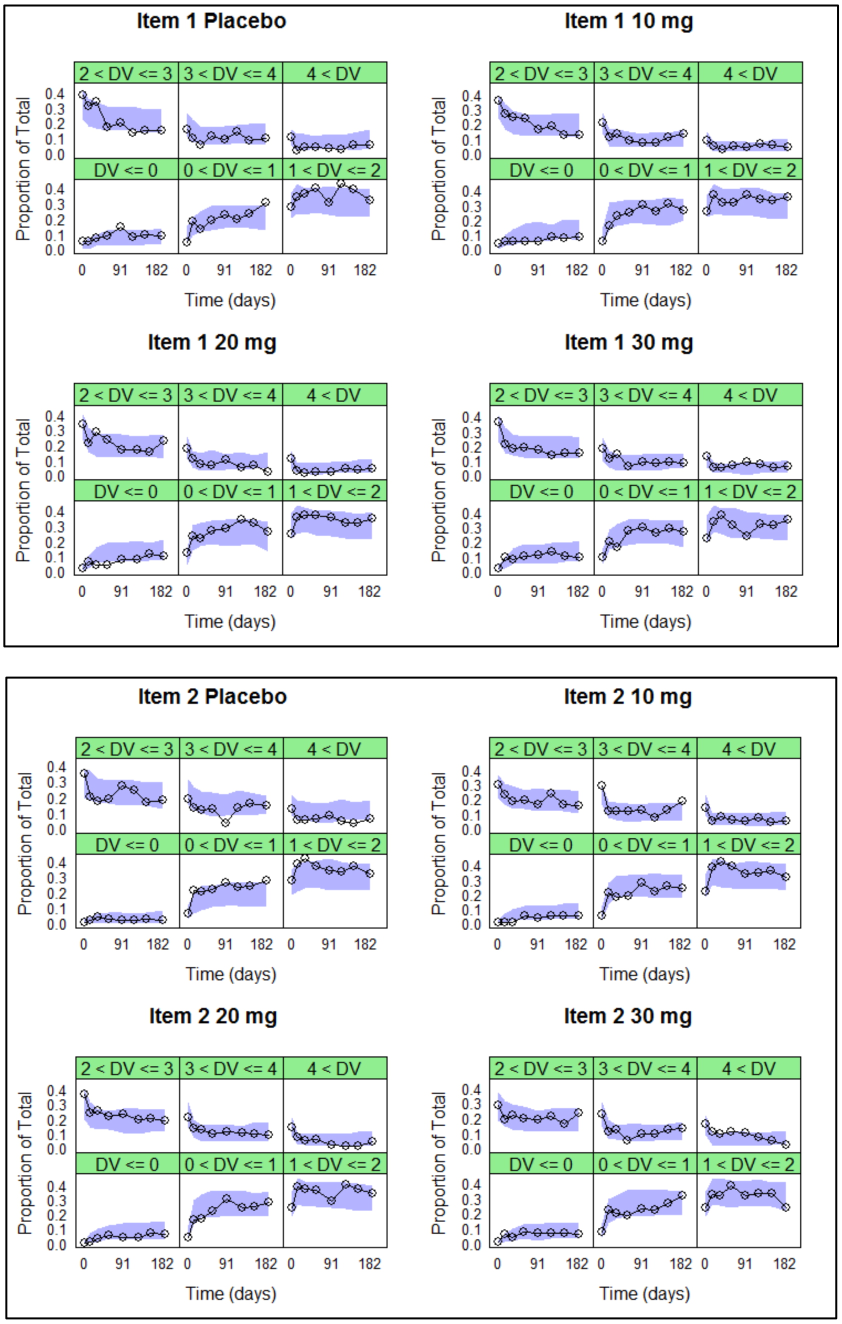

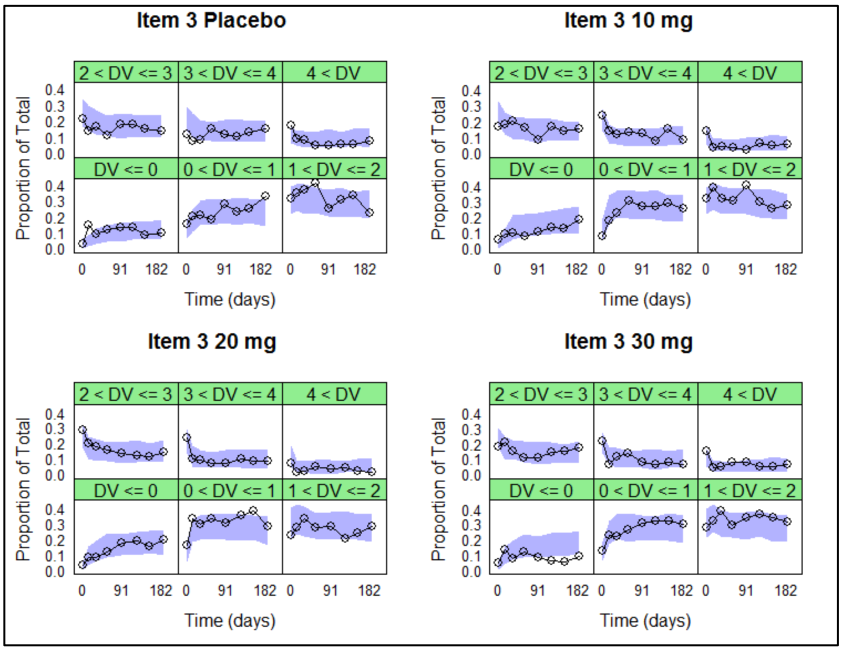
Item 3 Placebo
Item 3 10 mg
Item 3 20 mg
Item 3 30 mg
2 < DV <= 3
3 < DV <= 4
4 < DV
DV <= 0
0 < DV <= 1
1 < DV <= 2
Proportion of Total
Time (days)

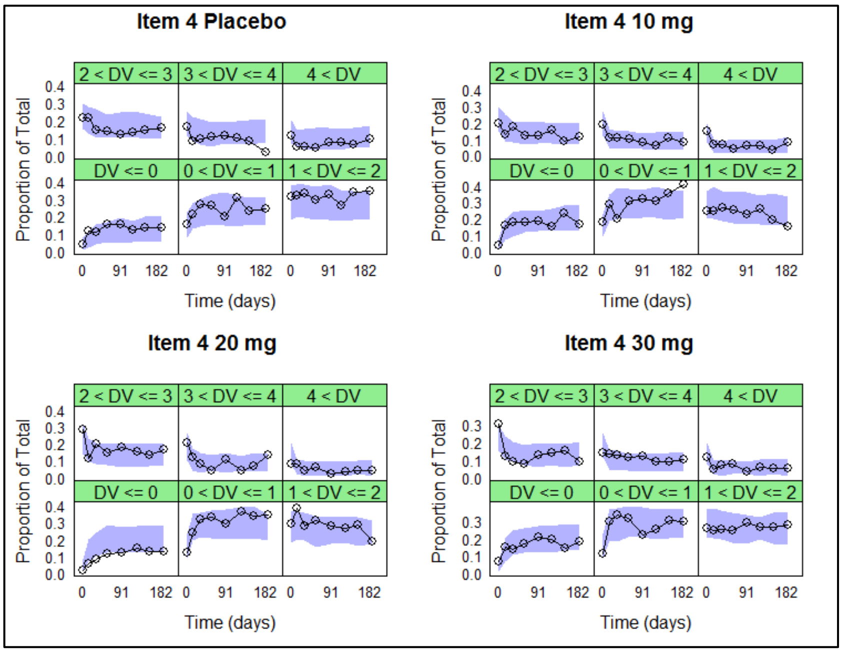
Item 4 Placebo
Item 4 10 mg
Item 4 20 mg
Item 4 30 mg
2 < DV <= 3
3 < DV <= 4
4 < DV
DV <= 0
0 < DV <= 1
1 < DV <= 2
Proportion of Total
Time (days)

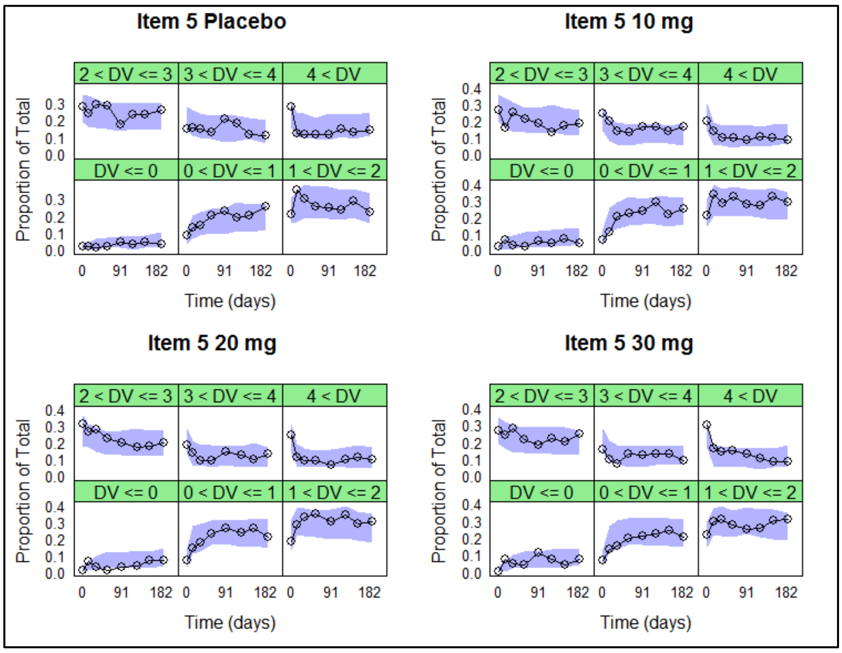
Item 5 Placebo
Item 5 10 mg
Item 5 20 mg
Item 5 30 mg
2 < DV <= 3
3 < DV <= 4
4 < DV
DV <= 0
0 < DV <= 1
1 < DV <= 2
Proportion of Total
Time (days)
0
91
182
0.0
0.1
0.2
0.3
0.4

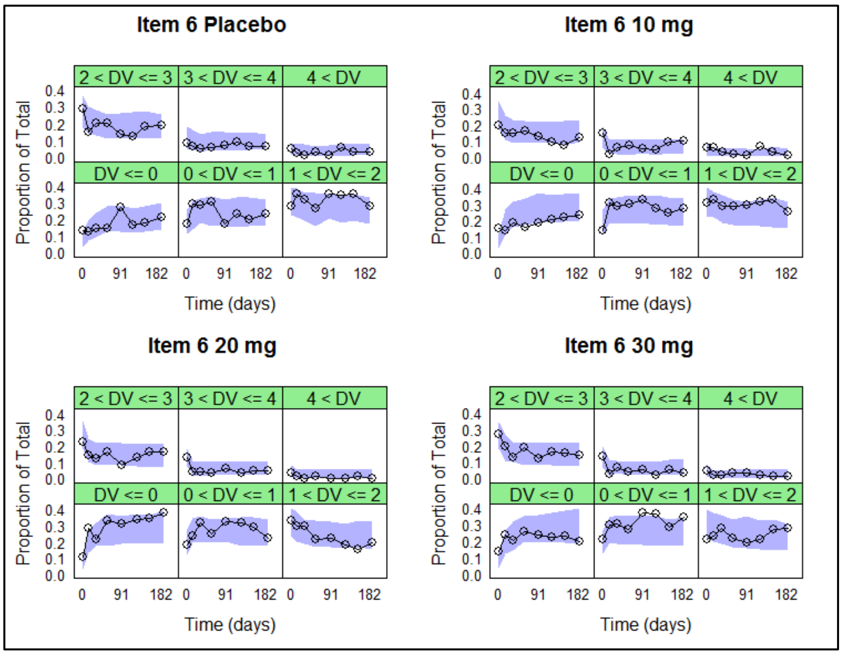
Item 6 Placebo
Item 6 10 mg
Item 6 20 mg
Item 6 30 mg
2 < DV <= 3
3 < DV <= 4
4 < DV
DV <= 0
0 < DV <= 1
1 < DV <= 2
Proportion of Total
Time (days)
0
91
182
0.0
0.1
0.2
0.3
0.4

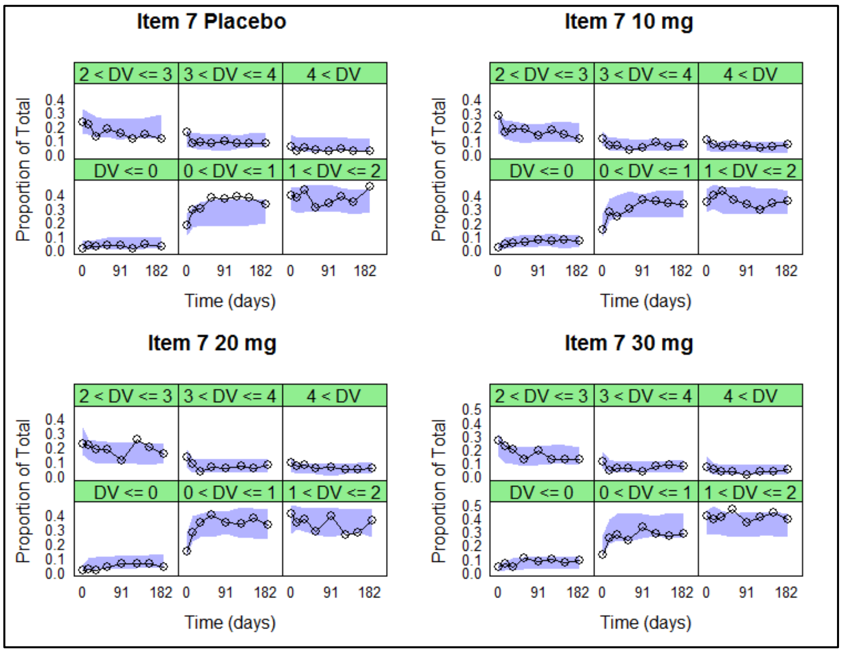

Supplemental Figure S13 – Item-level visual predictive checks in the longitudinal bidimensional item response theory IPSS model stratified by treatment arm. The observed frequency of each score over time is shown as points and the shaded areas indicate the 95% confidence intervals of the frequencies of each score in 200 simulated datasets. DV: dependent variable, i.e. observed score. IPSS: International Prostate Symptom Score.

12. **Supplemental Table S1**: Confirmatory Factor Analysis - Item factor loadings

Supplemental Table S1 – Item factor loadings in the confirmatory factor analysis with one and two factors, respectively, using Varimax orthogonal rotation. Using two factors, IPSS items 1, 3, 5, and 6 were mainly reflected by the first factor while items 2, 4, and 7 were mainly reflected by the second factor. Numbers in bold highlight the highest loading value for each IPSS item. IPSS: International Prostate Symptom Score.

IPSS item	Factor loadings using one factor	Factor loadings using two factors	
		Factor #1	Factor #2
Q1: Incomplete Emptying	0.780	**-0.634**	-0.422
Q2: Frequency	0.647	-0.215	**-0.866**
Q3: Intermittency	0.764	**-0.720**	-0.312
Q4: Urgency	0.624	-0.320	**-0.611**
Q5: Weak Stream	0.700	**-0.654**	-0.293
Q6: Straining	0.652	**-0.762**	-0.110
Q7: Nocturia	0.388	-0.159	**-0.434**

13. **Supplemental Table S2**: Parameter estimates of simultaneous longitudinal unidimensional item response theory model

Supplemental Table S2 – Parameter estimates in the longitudinal unidimensional item response theory International Prostate Symptom Score (IPSS) model estimating item characterstic curves and longitudinal parameters simultaneously. a_i is the discrimination parameter for item i, $b_{i,k}$ is the difficulty parameter for item i and category k. Item #1: "Incomplete Emptying"; Item #2: "Frequency"; Item #3: "Intermittency"; Item #4: "Urgency"; Item #5: "Weak Stream", Item #6: "Straining", Item #7: "Nocturia". BII: Benign Prostate Hyperplasia Impact Index, QoL: Quality of Life.

Parameter	Estimate	Relative standard error
IRT ICC parameters		
a_1	0.995	15.5%
$b_{1,1}$	-5.64	13.2%
$b_{1,2}$	2.5	14.8%
$b_{1,3}$	2.26	14.6%
$b_{1,4}$	1.96	14.7%
$b_{1,5}$	1.8	14.9%
a_2	0.73	15.3%
$b_{2,1}$	-7.31	12.9%
$b_{2,2}$	3.56	15.1%
$b_{2,3}$	2.72	14.3%
$b_{2,4}$	2.02	14%
$b_{2,5}$	2.12	15.3%
a_3	0.878	16.7%
$b_{3,1}$	-5.29	14%
$b_{3,2}$	2.54	15.9%
$b_{3,3}$	2.23	15.4%
$b_{3,4}$	1.52	15.8%
$b_{3,5}$	1.93	16.2%
a_4	0.73	15.9%
$b_{4,1}$	-5.15	12.7%
$b_{4,2}$	2.72	15.1%
$b_{4,3}$	2.18	14.4%
$b_{4,4}$	1.61	14.3%
$b_{4,5}$	1.87	15.2%
a_5	0.79	16.2%
$b_{5,1}$	-7.06	14.1%
$b_{5,2}$	3.19	15.9%
$b_{5,3}$	2.29	15.2%
$b_{5,4}$	1.8	15.3%
$b_{5,5}$	1.56	15.4%
a_6	0.672	17.4%
$b_{6,1}$	-4.32	14.3%
$b_{6,2}$	2.39	16.7%
$b_{6,3}$	2.33	16.5%
$b_{6,4}$	2.34	17.6%
$b_{6,5}$	2.38	18.9%
a_7	0.362	15.6%
$b_{7,1}$	-10.8	13.7%
$b_{7,2}$	7.04	15.9%
$b_{7,3}$	4.78	15.1%
$b_{7,4}$	3.33	15.6%
$b_{7,5}$	2.85	18.1%
Longitudinal parameters		
Baseline	0 (fixed)	
Pmax (maximal placebo response)	-1.42	19.1%
Tprog (placebo half-life)	12.2	20.9%
Drug effect	-0.732	22.3%
Covariates		
Baseline Box-Cox shape	0.301	32.2%
Baseline BII on Baseline	0.166	21.2%

Baseline QoL on Baseline	0.451	23.9%
-0.0803	-0.47	38.9%
Interindividual variability (IIV)		
IIV Baseline	100% (fixed)	
IIV Pmax	125.7%	15.4%
IIV Drift	1%	16.1%
IIV Tprog	52.2%	9.7%
IIV Pmax-Drift correlation	36%	

14. **Supplemental Table S3**: Stochastic simulation and estimation type I error

Supplemental Table S3 – Actual type I error rate used to inform the stochastic simulation and estimation procedure. One thousand data sets under each sample size were simulated from the bidimensional item response theory model without drug effects. The type I error was determined as the proportion of times the ΔOFV exceeded 3.84. The actual ΔOFV threshold (last column to the right) was derived empirically as the fifth percentile of the distribution of ΔOFVs in descending order. IPSS: International Prostate Symptom Score. IRT: Item response theory. df = degrees of freedom. ΔOFV: change in objective function value (reduced model OFV minus the full model OFV).

**Model (Total simulated trial sample size)**	**Actual type I error corresponding to a nominal type I error of 5%**	**df**	**ΔOFV corresponding to the actual type I error**
Total IPSS (N=33)	10.3%	1	6.03
Unidimensional IRT (N=33)	8.6%	1	5.27
Bidimensional IRT (N=33)	7.6%	2	6.98
Total IPSS (N=66)	6.0%	1	4.21
Unidimensional IRT (N=66)	4.7%	1	3.67
Bidimensional IRT (N=66)	3.9%	2	5.64
Total IPSS (N=99)	6.3%	1	4.81
Unidimensional IRT (N=99)	5.4%	1	4.03
Bidimensional IRT (N=99)	6.7%	2	6.57
Total IPSS (N=137)	5.4%	1	3.93
Unidimensional IRT (N=137)	5.4%	1	3.91
Bidimensional IRT (N=137)	6.1%	2	6.3

15. **Supplemental Discussion**

In the item characteristic curve (ICC) generalized additive model (GAM) smooth, η-shrinkage may cause the goodness-of-fit of the ICCs to appear worse than it actually is, and hence the traditional GAM smooth was expanded by incorporating random sampling from individual post-hoc η distributions, representing individual disability estimates and their uncertainty. Although the typical η-shrinkage and 95% CI of the individual shrinkage were below 10%, a substantially better fit was observed with the sampling-based method. This may imply that high individual η-shrinkage in the disability estimates of a small number of subjects in the population may lead to an inaccurate assessment of the visual goodness-of-fit of ICCs, and should be taken into account. In the current unidimensional IRT model, less than 10% of subjects had an individual η-shrinkage larger than 15% and less than 3% had an individual η-shrinkage larger than 20% (data not shown). Individuals with extreme values of the latent variable are likely to display the highest shrinkage, and it can be seen from **Figure 3** that it is especially at the tail ends of the disability distribution that the traditional GAM smooth underperforms compared to the developed sampling-based method.

An asymptotic exponential model with a drift parameter best described the longitudinal placebo data on both the total IPSS and latent disability scale. This model allowed patients' disease state to improve, worsen, or remain stable over time. Similar models have previously described the Young Mania Rating Scale (YMRS) score in bipolar disorder[1] and changes in the Hamilton Depression Rating Scale (HAMD-17)[2]. A Gompertz model has previously been used to describe longitudinal summary IPSS data[3]. However, a Gompertz model did not describe the data as well as the current model, and this may be due to differences in trial size and duration, in placebo effects (oral vs. subcutaneous administration), and differences in patient population characteristics.

Covariate analysis identified baseline QoL and baseline BII to explain variability in the *Baseline* parameter in both the longitudinal summary IPSS model as well as the longitudinal unidimensional IRT model. This finding is supported by several studies that have shown a high correlation between IPSS and QoL[4], IPSS and BII[5–7], as well as Qol and BII[7–10]. Moreover, in the current trial, the IPSS and latent disability baselines were significantly higher in patients from North America compared to patients from Europe, potentially rooted in differences in lifestyle-related factors. The similarity between included covariate relationships in the longitudinal IPSS and IRT models, respectively, is expected due to the high correlation between disability estimates and observed IPSS (**Figure 3**). Lastly, the baseline QoL score was found to explain variability in maximal IPSS placebo response, indicating that the worse patients perceive their QoL at baseline, the larger their ensuing placebo response on the IPSS scale. As a high number of covariate relationships were investigated in the current study, it was not possible to implement a full covariate model approach[11], as this would affect model stability. An advantage of stepwise covariate search is that it allows for automated screening of numerous covariate relationships. It is however to be noted that

this approach relies on statistical signifance rather than clinical importance or explanatory power of ultimately included covariates.

In the current work, we showed that the power of the ANCOVA using the WOT strategy is higher compared to the ANCOVA considering only the landmark time point. By taking the average of the change from baseline over several visits, the within-subject variability, and consequently, the total variability in the group means, is reduced. This allows for higher power to identify significant treatment effect. As the simulated data did not include any drop-out, the use of the WOT strategy is appropriate as the average was based on an equal number of measurements in the treatment period from all patients. The pharmacometric models all displayed higher power compared to the WOT ANCOVA, and this comparison has to our knowledge not been presented beforehand. This higher power mainly stems from the use of longitudinal individual patient data in a nonlinear mixed effects modeling framework compared to averaging the longitudinal data within patients, the latter leading to a loss of information and lower power.

References

1. Sun W, Laughren TP, Zhu H, Hochhaus G, Wang Y. Development of a placebo effect model combined with a dropout model for bipolar disorder. J Pharmacokinet Pharmacodyn. 2013 Jun;40(3):359–68.

2. Gomeni R, Merlo-Pich E. Bayesian modelling and ROC analysis to predict placebo responders using clinical score measured in the initial weeks of treatment in depression trials. Br J Clin Pharmacol. 2007 May;63(5):595–613.

3. D'Agate, S. PAGE 2018 III-77 Development of a drug-disease model describing individual IPSS trajectories in BPH patients: Implication of disease progression and covariate factors on long term treatment response.

4. O'leary MP. Validity of the "bother score" in the evaluation and treatment of symptomatic benign prostatic hyperplasia. Rev Urol. 2005;7(1):1–10.

5. Barry MJ, Fowler FJ, O'Leary MP, Bruskewitz RC, Holtgrewe HL, Mebust WK, et al. The American Urological Association symptom index for benign prostatic hyperplasia. The Measurement Committee of the American Urological Association. J Urol. 1992 Nov;148(5):1549–57; discussion 1564.

6. Barry MJ, Williford WO, Chang Y, Machi M, Jones KM, Walker-Corkery E, et al. Benign prostatic hyperplasia specific health status measures in clinical research: how much change in the American Urological Association symptom index and the benign prostatic hyperplasia impact index is perceptible to patients? J Urol. 1995 Nov;154(5):1770–4.

7. Angalakuditi M, Seifert RF, Hayes RP, O'Leary MP, Viktrup L. Measurement properties of the benign prostatic hyperplasia impact index in tadalafil studies. Health Qual Life Outcomes. 2010 Nov 12;8:131.

8. Desgrandchamps F, Droupy S, Irani J, Saussine C, Comenducci A. Effect of dutasteride on the symptoms of benign prostatic hyperplasia, and patient quality of life and discomfort, in clinical practice. BJU Int. 2006 Jul;98(1):83–8.

9. O'Leary MP, Wei JT, Roehrborn CG, Miner M, BPH Registry and Patient Survey Steering Committee. Correlation of the International Prostate Symptom Score bother question with the Benign Prostatic Hyperplasia Impact Index in a clinical practice setting. BJU Int. 2008 Jun;101(12):1531–5.

10. Boyle P, Robertson C, Mazzetta C, Keech M, Hobbs R, Fourcade R, et al. The relationship between lower urinary tract symptoms and health status: the UREPIK study. BJU Int. 2003 Oct;92(6):575–80.

11. Gastonguay MR. A Full Model Estimation Approach for Covariate Effects: Inference Based on Clinical Importance and Estimation Precision. 2004.
